# Computational drug repositioning approach to predict multi-target therapeutics for epilepsy

**DOI:** 10.1038/s41598-025-27625-2

**Published:** 2025-12-16

**Authors:** Pawan Kumar, Vivek Kumar, Raveena Chauhan, Vandana Saini, Ajit Kumar

**Affiliations:** 1 Toxicology and Computational Biology Group, Centre for Bioinformatics, Maharshi Dayanand University, Rohtak, Haryana 124001 India; 2 Department of Bioinformatics & Computational Biology, College of Biotechnology, CCS Haryana Agricultural University, Hisar, Haryana 125004 India

**Keywords:** AED, Drug repurposing/Repositioning, Epilepsy, Molecular docking, Molecular dynamic simulations, Seizures, Computational biology and bioinformatics, Computational neuroscience, Virtual drug screening

## Abstract

Epilepsy affects millions of people globally, with approximately one-third of patients experiencing drug-resistant seizures. Developing new anti-epileptic drugs is time-intensive and costly, prompting interest in computational drug repositioning strategies. Here, we report on a comprehensive drug repositioning approach to identify the multi-targeted therapeutic option(s) for epileptic seizures. All approved drugs from the DrugBank database were screened for their anti-epileptic properties, which involved predicting their blood-brain permeability and clustering them based on structural similarity with marketed anti-epilepsy drugs. The screened drugs were subjected to molecular docking against previously identified therapeutic target proteins (Voltage-Gated Sodium Channel α2; GABA receptor α1-β1; and Voltage-Gated Calcium Channel α1G), A total of 46 drugs showed better binding affinity than the respective standard drugs - Carbamazepine, Clonazepam and Pregabalin for the selected target proteins - Voltage-Gated Sodium Channel α2; GABA receptor α1-β1; and Voltage-Gated Calcium Channel α1G, respectively. The binding pocket and literature data mining revealed three drugs, Oxaprozin, Pizotifen, and Cyproheptadine, that bind within the precise binding pocket and have no reported severe side effects related to seizure onset. The molecular dynamics simulation studies revealed that all three compounds exhibited more stable and better binding interactions with their corresponding drug targets. Oxaprozin, among the identified three drugs, showed a very stable binding and can be considered a potential repurposed drug for epilepsy, warranting further preclinical trials.

**Supplementary Information:**

The online version contains supplementary material available at 10.1038/s41598-025-27625-2.

## Introduction

Epilepsy is one of the most prevalent neurological disorders globally, affecting millions of individuals of all ages^1,2^. It encompasses a spectrum of neurological disorders characterised by abnormal electrical activity in the brain, leading to seizures. These seizures can manifest in various forms, ranging from momentary lapses of consciousness to convulsions. The underlying causes of epilepsy are diverse, including genetic predispositions, brain injuries, infections, and developmental abnormalities^3,4^. Epilepsy poses significant challenges to patients’ quality of life, societal integration, and healthcare systems worldwide. Numerous anti-epileptic drugs (AEDs) are available, but a substantial proportion of epilepsy patients experience inadequate seizure control or intolerable side effects with standard medications^5^.

Furthermore, the development of novel AEDs requires considerable time, resources, and navigates regulatory obstacles, encompassing comprehensive clinical trials, thorough safety assessments, and intricate approval procedures, which can considerably postpone their accessibility to patients, rendering it a formidable challenge. Consequently, alternative approaches, such as drug repositioning, which entails identifying new therapeutic applications for existing medications, become essential. This approach has the potential to accelerate the discovery and endorsement of efficacious treatments by circumventing numerous preliminary development phases and utilising established safety profiles.

Drug repositioning, also known as drug repurposing or reprofiling, involves identifying new therapeutic applications for existing drugs beyond their initially intended indications^6^. Unlike traditional drug discovery approaches, which often start from scratch, drug repositioning leverages existing pharmacological data, clinical experience, and safety profiles of approved drugs. This strategy offers several advantages, including reduced development costs, shorter timelines, and an increased likelihood of success in clinical trials^7,8^. Recent years have witnessed a growing interest in exploring drug repositioning strategies for epilepsy treatment, resulting in systematic screening of approved drug libraries and investigational compounds to identify candidates with anti-epileptic properties. These efforts have yielded promising findings, as the repurposed drug Lorcaserin^9^ demonstrates its efficacy in preclinical models and is currently under early-stage clinical trials for epilepsy.

The present study employed a virtual high-throughput screening (VHTS)-based, multi-target drug repositioning strategy to identify drug compounds with anti-epileptic activity. The DrugBank database was selected to screen approved drug compounds based on blood-brain barrier (BBB) permeability, structural similarity, molecular docking and binding site analysis. Drug(s) showing better binding affinity were evaluated for binding stability and free binding energy using molecular dynamics simulation (MDS) for 100 ns.

## Materials and methodology

### Retrieval of structural and physicochemical data of drug molecules

The DrugBank database is one of the most comprehensive structural databases, containing chemical, physicochemical, pharmacokinetic, pharmacodynamic, target, and metabolomic information on marketed drugs, including approved small-molecule drugs, biotech drugs, investigational drugs, and withdrawn drugs^10^. Due to safety concerns, experimental and withdrawn drug compounds were not considered, while approved drugs, including illicit and nutraceutical drugs, were selected for further in-silico screening. The 3D molecular files for all drug compounds were retrieved from the DrugBank database’s File Transfer Protocol (FTP) server as a single Structure Data File (SDF).

### Screening of drugs for BBB permeability

The Central Nervous System (CNS) acting drug compounds must cross the blood-brain barrier (BBB) to reach the neural system for pharmacological activity. In-vivo or in-vitro BBB permeability prediction is a complex, time- and cost-intensive process. So, as an alternative, Machine Learning (ML) based in-silico BBB permeability prediction is a time-efficient and economical process with superior accuracy. Different BBB permeability prediction tools are available with great accuracy, but to gain maximum positive results, we used three different tools: (a) an *in-house* developed tool, BBBper (Blood Brain Barrier permeability prediction tool)^11^, AdmetSAR^12^ and LightBBB^13^ to screen the selected approved drug compounds for BBB permeability.

### Clustering of structurally similar drugs

Most studied phenomena of drug repositioning focus on the similarity among drugs’ structural fingerprints, stating that “similar structures will have similar activity”^8,14,15^. So, all selected BBB-permeable drugs were further filtered for their structural similarity with available marketed AEDs (Table 1)^16^. The ChemMine tool^17^, an online molecular data analysis program supported by the R library ChemMineR^18^, was used to cluster selected approved and BBB-permeable drugs using binning clustering applications. The binning clustering method is used to partition a dataset into clusters by quantising the data points into bins and further assigning each bin to a cluster. Different drug clusters were generated using different similarity cutoff values ranging from 0.4 to 0.9. A lower cutoff value results in a lower similarity between compounds, leading to the clustering of less similar compounds. In contrast, a higher similarity cutoff results in the clustering of more similar compounds, leading to smaller cluster sizes. Finally, the similarity cutoff, which grouped most marketed AEDs within a single cluster, was selected for grouping drug compounds with similar structures to marketed AEDs. All compounds within the selected AEDs cluster were chosen for subsequent molecular docking analysis, based on the hypothesis that their structural conformations were analogous to those of established AEDs and, therefore, may elicit comparable therapeutic effects.

**Table 1:** List of marketed anti-epileptic drugs.

Drug Name	DrugBank ID	Mechanism of action (from DrugBank)
Acetazolamide	DB00819	Carbonic anhydrase inhibitor.
Brivaracetam	DB05541	Synaptic vesicle glycoprotein 2 A (SV2A) agonist. VGSC alpha 1B inhibitor.
Cannabidiol	DB09061	Weak partial agonist activity at Cannabinoid receptors CB1R and CB2R. Inhibits noradrenaline, dopamine, serotonin and GABA uptake^19^. Block T-type (low voltage-activated) Ca channel^19^. Antagonise mu-opioid receptor.
Carbamazepine	DB00564	Inhibits VGSC alpha subuni^20^. Decrease dopamine turnover (dopamine antagonist) by reducing dopamine (D2) receptor density and phosphorylation. Enhance GABA synthesis. Inhibits Serotonin uptake. Decrease Norepinephrine release.
Clobazam	DB00349	GABA-A receptor partial agonist (alpha and gamma 2 subunits).
Clonazepam	DB01068	GABA-A receptor agonist^21^.
Diazepam	DB00829	GABA-A receptor agonist.
Dronabinol	DB00470	Cannabinoid receptors 1 and 2 agonists.
Eslicarbazepine acetate	DB09119	Inhibits VGSC^22^. Inhibits T-type calcium channel.
Ethosuximide	DB00593	Inhibits T-type VGCC alpha 1G^23^.
Ethotoin	DB00754	Inhibits VGSC alpha 5.
Ezogabine	DB04953	VGPC (Kv7.2-7.5) KCNQ2,3,4,5) agonist.
Felbamate	DB00949	Antagonise Glutamate receptor (NMDA 2 A,3 A, 2B).
FosPhenytoin	DB01320	Inhibits VGSC alpha 5.
Gabapentin	DB00996	Structural analogue of GABA^24^. Inhibits VGCC subunit alpha 2, delta 1,2. Activates VGPC subfamily KQT member 3,5.
Lacosamide	DB06218	Inhibits VGSC alpha 3,9,10^22,25^.
Lamotrigine (phenyl triazine)	DB00555	Inhibits VGSC^23^. Inhibits adenosine A1/A2 receptors. Inhibits the K-opioid receptor.
Levetiracetam	DB01202	Agonist for Synaptic vesicle glycoprotein 2 A (SV2A)^26^. Inhibits VGSC alpha 1B^27,28^.
Lorazepam	DB00186	GABA-alpha receptor agonist.
Methsuximide	DB05246	Inhibits VGCC T-type subunit alpha 1G.
Methylphenobarbital	DB00849	GABA-alpha receptor agonist. Glutamate receptor antagonist.
Midazolam	DB00683	GABA-alpha receptor agonist.
Nitrazepam	DB01595	GABA R agonist. Inhibits Voltage-dependent sodium channels.
Oxcarbazepine	DB00776	Inhibits VGSC^20,22^.
Perampanel	DB08883	Glutamate receptor 1 antagonist^29^.
Phenacemide	DB01121	Inhibits the Sodium channel protein type 1 subunit alpha
Phenobarbital	DB01174	GABA R agonist. Glutamate receptor antagonist. Inhibits Calcium channels. NMDA channel antagonist.
Phenytoin	DB00252	VGSC blocker alpha 1, 3 and 5 subunits.
Pregabalin	DB00230	Inhibits VGCC subunit alpha 2/ delta 1^30,31^.
Primidone	DB00794	GABA alpha receptor agonist. Glutamate receptor antagonist.
Rufinamide	DB06201	Inhibits VGSC^32^.
Stiripentol	DB09118	GABA alpha receptor agonist.
Tiagabine	DB00906	Inhibits GABA transferase.
Topiramate	DB00273	Inhibits VGSC type 1 alpha subunit^29^. GABA alpha 1 receptor agonist.
Trimethadione	DB00347	Inhibits Voltage-dependent T-type calcium channel subunit alpha-1G
Valproic acid	DB00313	Inhibits succinic semialdehyde dehydrogenase (SSADH)^33^. Inhibits VGSC^34,35^. Inhibits GABA transferase^33^. Inhibits Histone deacetylase 2 and 9.
Vigabatrin	DB01080	GABA analogue^36^. Irreversible inhibitor of 4-aminobutyrate transaminase. GABA beta receptor agonist^36^.
Zonisamide	DB00909	Inhibits VGSC alpha 1,2,3,4,5,9,11 subunits^37^. Inhibits VGSC beta 1,2,3,4 subunits^37^. Inhibits VGCC T-type subunit alpha 1G, 1 H, 1I. Inhibits Carbonic anhydrase 1,2,3,4,5 A,5B,6,7,10,11,1213,14.

### Selection and preparation of the tertiary structure of epilepsy target receptors

Previously, we identified three first-line epilepsy therapeutic targets: the Voltage-Gated Sodium Channel (VGSC) α2 (Nav1.2), the Gamma-Aminobutyric Acid (GABA) receptor α1-β1, and the Voltage-Gated Calcium Channel (VGCC) α1G (Cav3.1)^16^. The tertiary protein structures of the Nav1.2 receptor, the GABA receptor α1 subunit, and the Cav3.1 channel were available in the RCSB-PDB database, with PDB IDs 6J8E^38^, 6HUJ^39^, and 6KZP^40^, respectively. However, the tertiary structure of the GABA receptor β1 was unavailable in the RCSB-PDB database; therefore, its 3D structure was generated by homology modelling using the SWISS-MODEL web server^41^. The GABA receptor β1 fasta sequence was retrieved from UniProt (UniProt ID: P18505) and subjected to homology modelling using the Swiss Model web server, with 6HUJ-B as the template. The modelled structure was further validated using the QMEAN score^42^, Ramachandran plot^43^, Verify3D^44^, and ProSA Z-score^45^. To form the functional GABA receptor α1-β1 complex, the available GABA receptor α1 chain and modelled GABA receptor β1 chain were subjected to protein-protein docking using Hex tool v8.0.0^46^. A total of 25 searches were performed, using “shape + electro + DARS” as the correlation types and the 3D FFT mode. A side-by-side (parallel) conformation, showing head-to-head and tail-to-tail orientations for both chains, was selected, and the result was saved as a combined PDB file. The conformation of the generated GABA receptor α1-β1 file was also validated by aligning the generated structure against the GABA receptor α1-β3 (PDB ID: 6HUJ) using open-source PyMOL v2.5.0^47^.

All the selected epilepsy target receptor proteins (Nav1.2, GABA receptor α1-β1, and Cav3.1) were subjected to energy minimisation using UCSF Chimera v1.6^48^ for 100 steepest descent and 10 conjugate gradient steps under AMBER ff99bsc force field with a step size of 0.02Å. The energy-minimised structures were saved as PDB files for further molecular docking studies.

### Molecular Docking study

The selected epilepsy target receptor proteins (Nav1.2, GABA receptor α1-β1, and Cav3.1) were prepared for molecular docking by adding polar hydrogen and assigning Kollman and Gasteiger charges using Autodock tools. Autodock v4.2.6^49^ was used for molecular docking of selected drug compounds against specific therapeutic target proteins. The target proteins Nav1.2 and Cav3.1 are channel proteins, and to block the channel, the Grid file parameters (GPF) were assigned around the pore region, while the grid parameters for GABA receptor α1-β1 were assigned between the α1 and β1 chains within the GABA binding area to find a GABA agonist^50^ (Table 2). Molecular docking was performed for 100 independent runs using the Lamarckian genetic algorithm with a population size of 150, taking a gene mutation rate of 0.02 and a crossover rate of 0.8. The dock conformation with the lowest binding energy and maximum cluster size was selected for each drug. The highest-selling AEDs, Carbamazepine, Clonazepam, and Pregabalin, were chosen as standard drugs for targeting the proteins Nav1.2, GABA receptor α1-β1, and Cav3.1, respectively^16^. The drugs showing better binding affinities than the corresponding standard AEDs against all three receptors were selected for further study.

**Table 2:** Grid box parameters for molecular Docking study of voltage-gated sodium channel 2 A (Nav1.2), GABA receptor α1-β, and voltage-gated calcium channel α1G (Cav3.1).

	Nav1.2	GABA Receptor α1-β1	Cav3.1
Size-X	54	56	64
Size-Y	66	76	76
Size-Z	94	76	90
Center-X	129.988	119.412	176.584
Center-Y	132.695	134.518	168.642
Center-Z	135.591	159.123	192.98
Grid Box			

### Binding pocket analysis of selected drugs

The residues within the binding pockets are significant factors in a drug’s pharmacodynamic effect. Hence, the binding pockets of the selected drugs were compared with those of the standard drugs, hypothesising that drugs with binding pockets similar to those of the standard AEDs would produce similar therapeutic effects. Therefore, the dock complexes of standard AEDs and selected drugs against the epilepsy target proteins were generated using the Autodock tool, and the binding pockets (nearby amino acids and hydrogen bond-forming amino acids) for each dock complex were analysed using the Java-based tool LigPlot+^51^. Drugs showing similar binding pockets to the standards and better binding affinities than corresponding standard drugs would have similar but better therapeutic effects than the existing standard/marketed drugs, and were hence selected for further repositioning study.

### Literature data mining

Selected drugs with better binding affinities and similar binding pockets to their corresponding standard drugs were analysed for previous reports regarding epilepsy/seizure or any other severe side effects related to suicidal thinking, abnormal heartbeats, etc. Drugs showing any seizure-persuading or severe side effects cannot serve as a potential repositioned drug candidate. Therefore, drugs with no report or any prior study on seizure reduction with acceptable side effects were selected for further repositioning study.

### Molecular dynamics simulation study

Selected repositioned drugs with better binding energies than corresponding standard drugs against selected epilepsy target receptors were subjected to Molecular Dynamics Simulation (MDS) using GROMACS v2022.1^52^. The charmm36m force field^53^ was used to generate topology files for the docked complex of the selected drugs and the epilepsy receptor(s). The entire system was solvated with TIP3P water molecules in a rectangular box, followed by the addition of sodium (Na+) and chloride (Cl-) ions to neutralise the system at a concentration of 0.15 M, to mimic physiological conditions. To relieve the system’s geometric strain, a maximum of 5,000 energy minimisation steps were performed using the steepest descent algorithm to lower the potential energy up to 1,000 KJ/mol. Then, the whole system was equilibrated under constant temperature (310 K) and pressure (1 bar) conditions for 1000 ps (1 ns) using the Nose-Hoover thermostat and Parrinello-Rahman barostat, respectively. After equilibration, MDS was performed for 100 ns (500,000,000 steps) in triplicate with a time step of 2 fs at constant temperature and pressure using Periodic Boundary Conditions (PBC). Trajectories were recorded every 100 ps. Protein-drug interactions throughout the whole simulation were monitored for stability examination.

The obtained trajectory of the simulated protein-ligand complexes was analysed based on Root Mean Square Deviation (RMSD), Root Mean Square Fluctuations (RMSF), Radius of gyration (Rg), Solvent Accessible Surface Area (SASA), Principal Component Analysis (PCA), interaction energy including Lennard-Jones potential and Coulombic interactions, and Hydrogen bond analysis. Further MMPBSA was performed using gmx_MMPBSA^54^ to calculate the free binding energy between the protein and drug for the entire MDS.

## Results and discussion

### Retrieval of approved drugs from the drugbank database

**Fig. 1:**
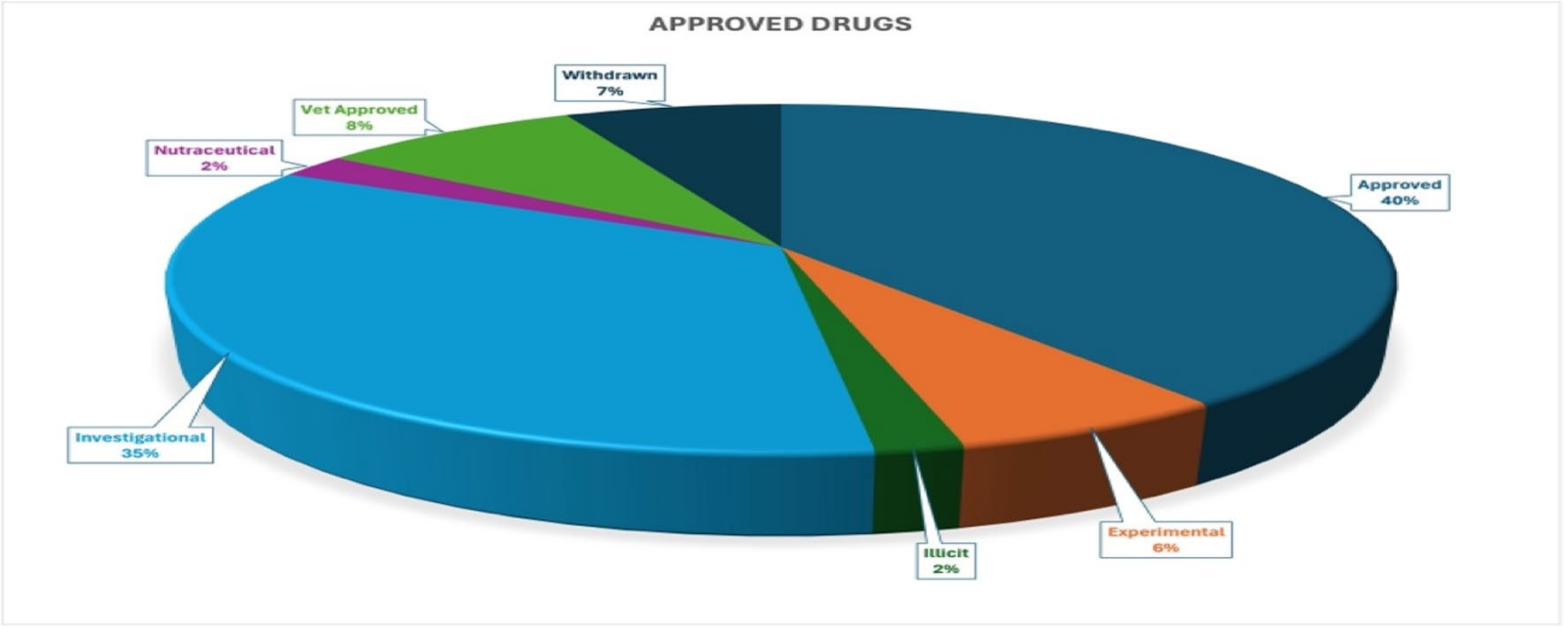
Approved drug classification at the DrugBank database.

The DrugBank database v5.1.12 (accessed January 1, 2025) contained structural and physicochemical data for 12,699 drugs, including approved, illicit, nutraceuticals, investigational, experimental, veterinary, and withdrawn drugs (Supplementary File: S1). For safety purposes, only approved drugs (2,769) were considered for our drug repositioning study (Fig. 1; Supplementary File: S2; Table 3). Among these, 188 drugs were found to be withdrawn from the market after their initial approval. Hence, the remaining 2,581 approved drugs were selected for further drug repositioning screening as multitarget anti-epileptic therapeutics. The structural (SDF) and physicochemical data for the selected 2581 drugs were retrieved using the FTP service of the DrugBank database. The selected approved drugs also included 38 marketed AEDs (Table 1), which were used for structural similarity analyses.

**Table 3:** Classification of approved drugs from the drugbank database.

Drug groups	Number
Approved	1099
Approved; Experimental	152
Approved; Experimental; Investigational	22
Approved; Experimental; Investigational; Withdrawn	1
Approved; Experimental; Vet Approved	2
Approved; Experimental; Withdrawn	1
Approved; Illicit	39
Approved; Illicit; Investigational	12
Approved; Illicit; Investigational; Vet Approved	4
Approved; Illicit; Investigational; Withdrawn	5
Approved; Illicit; Vet Approved	1
Approved; Illicit; Withdrawn	5
Approved; Investigational	963
Approved; Investigational; Nutraceutical	24
Approved; Investigational; Nutraceutical; Vet Approved	3
Approved; Investigational; Nutraceutical; Withdrawn	1
Approved; Investigational; Vet Approved	77
Approved; Investigational; Vet Approved; Withdrawn	8
Approved; Investigational; Withdrawn	57
Approved; Nutraceutical	36
Approved; Nutraceutical; Vet Approved	10
Approved; Nutraceutical; Withdrawn	1
Approved; Vet Approved	137
Approved; Vet Approved; Withdrawn	15
Approved; Withdrawn	94
Total	2769

### Screening of drugs for BBB permeability

**Fig. 2:**
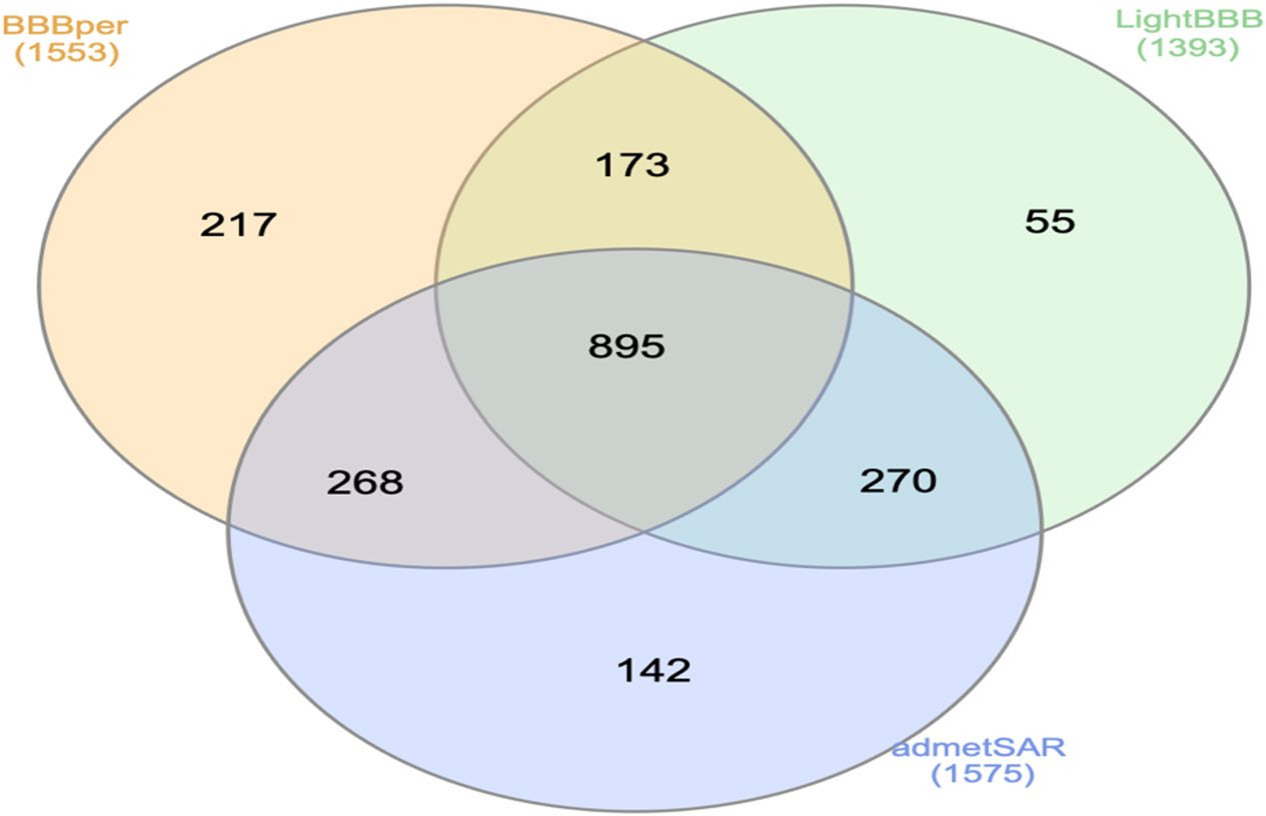
Drugs predicted to permeate the Blood-Brain-Barrier using BBBper, LightBBB, and admetSAR.

AEDs must cross the BBB to perform their action within the CNS. Three different ML algorithm-based BBB permeability prediction programs/tools were used for better accuracy. Our *in-house* developed tool, BBBper^11^, predicted 1553 BBB-permeable drugs, while admetSAR^12^ predicted the maximum number of 1575 drugs as BBB-permeable, and LightBBB^13^ predicted 1393 drugs to cross the BBB (Supplementary File: S3). All three selected programs/tools predicted 895 drugs as BBB-permeable (Fig. 2). BBBper predicted all 38 AEDs as BBB permeable, while admetSAR and LightBBB showed 37 AEDs as BBB permeable, and FosPhenytoin and Eslicarbazepine as BBB impermeable, respectively. Hence, to mitigate adverse selection, drug compounds predicted to be BBB-permeable by at least two programs/tools were selected for further study. The selection criteria resulted in 1606 drug compounds (including 38 marketed AEDs) as BBB-permeable.

### Clustering of structurally similar drugs

**Fig. 3:**
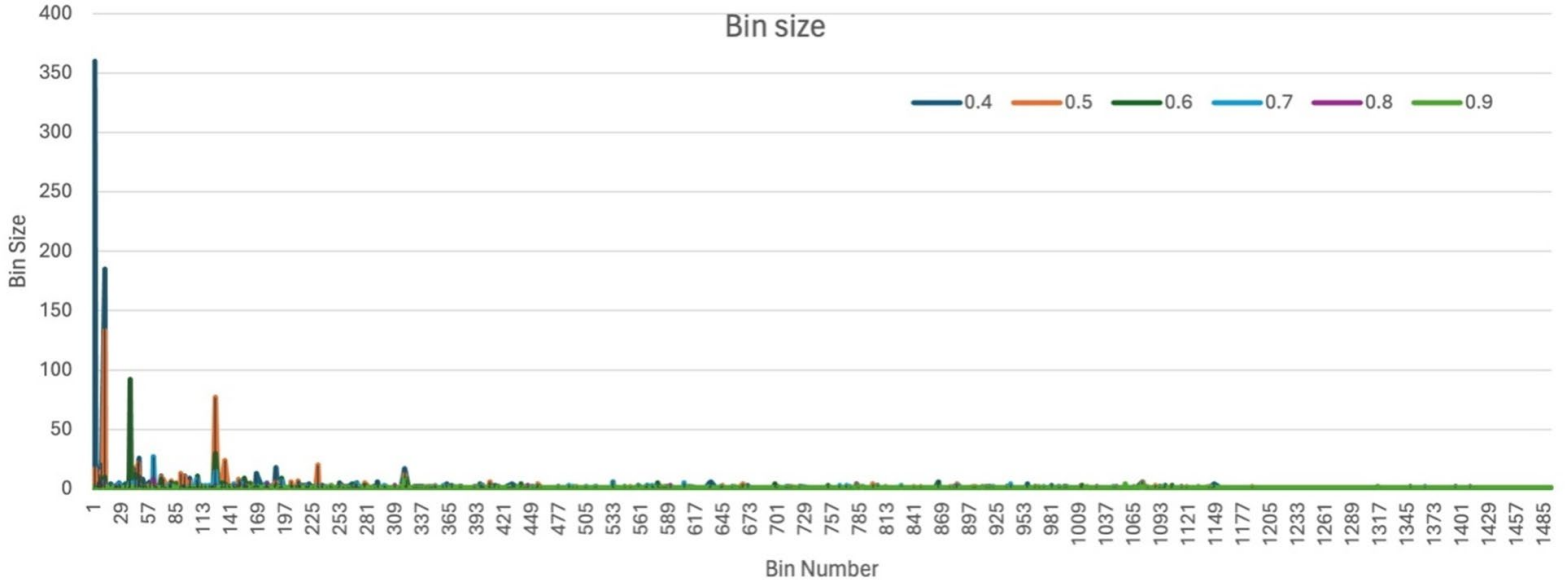
Bin size observed for drug compounds at different cutoff values using the ChemMine tool.

To identify structurally similar drugs, the selected BBB-permeable drugs were clustered based on structural similarity using the ChemMine tool^17^. Different similarity cutoff values, specifically 0.4, 0.5, 0.6, 0.7, 0.8, and 0.9, resulted in the formation of 645, 927, 1128, 1296, 1449, and 1492 bins, respectively (Supplementary File: S4). At a lower cutoff value, such as 0.4, the clustering produces fewer, larger bin counts, indicating broader grouping criteria; some bins include hundreds of compounds (bin-2: 360; bin-12: 185), encompassing most AEDs (bin-2: 18 AEDs). As the cutoff increases to 0.5 and 0.6, the number of bins rises, and their sizes decrease (bin-12: 133; bin-38: 92), reflecting more stringent similarity thresholds that lead to more refined subdivisions of the drug dataset. Beyond a cutoff of 0.6, minimal changes in bin sizes suggest a plateau in clustering resolution, indicating that further increasing the cutoff may not significantly improve cluster differentiation (Fig. 3).

Bin-2, at the cutoff value of 0.4, was observed to have the highest number (18) of AEDs, including Carbamazepine, Clobazam, Clonazepam, Diazepam, Eslicarbazepine, Ethotoin, FosPhenytoin, Lorazepam, Methylphenobarbital, Midazolam, Methsuximide, Nitrazepam, Oxcarbazepine, Perampanel, Phenacemide, Phenobarbital, Phenytoin, and Primidone. Therefore, bin-2, with a cutoff value of 0.4, was chosen for further study because it combines a high drug count with a structural similarity of at least 40%, aligning well with marketed AED profiles. Along with bin-2, other bins at the same cutoff (0.4) contain only a single AED, highlighting a high degree of structural specificity. For example, bins like Bin-65 (Pregabalin), Bin-96 (Topiramate), Bin-120 (Valproate), Bin-286 (Ethosuximide), Bin-475 (Tiagabine), Bin-477 (Zonisamide), Bin-501 (Felbamate), Bin-531 (Gabapentin), Bin-578 (Vigabatrin), Bin-824 (Ezogabine), Bin-855 (Rufinamide), Bin-863 (Lacosamide) and Bin-1029 (Stiripentol), each contain only one drug, representing the unique structure of these 13 marketed AEDs within the dataset. Conversely, some bins, such as Bin-213 and Bin-649, contain multiple drugs, including a pair of AEDs (Bin-213: Cannabidiol and Dronabinol, and Bin-649: Brivaracetam and Levetiracetam), along with one other structurally similar drug. Additionally, Bin-140 and Bin-348 feature two drugs each, one AED (Bin-140: Trimethadione; and Bin-348: Acetazolamide) and one structurally similar drug compound, illustrating potential structural relationships. In Bin-54, Lamotrigine was observed with three other structurally similar drug compounds. Overall, 349 drug compounds with over 40% structural similarity to marketed AEDs were identified across all these bins. All these compounds were considered for further repositioning studies.

### Selection and Preparation of the tertiary structure of epilepsy target receptors

Three epilepsy drug targets, VGSC α2 (Nav1.2), GABA receptor α1-β1, and VGCC α1G (Cav3.1), were reportedly identified as first-line therapy targets for epilepsy^16^. The tertiary protein structures of the Nav1.2 receptor, the GABA receptor α1 subunit, and the Cav3.1 channel were available in the RCSB-PDB database with PDB IDs 6J8E-A, 6HUJ-A, and 6KZP, respectively. The tertiary structure of the GABA receptor β1 (UniProt ID: P18505) was not available in the RCSB-PDB database. Furthermore, a sequential similarity of 54% was observed between GABA receptor β1 and β3, with a query coverage of 78.28%, making it a suitable choice for homology modelling. Using the SwissModel web server, a homology model of the GABA receptor β1 was generated, with PDB ID 6HUJ-B of the GABA receptor β3 serving as a template. The modelled 3D structure was evaluated with a QMEAN score of -2.88, a Ramachandran Z-score of -2.818, and a pass verify-3D status. Ramachandran plot analysis revealed that 93.4% of the amino acids reside under the favoured region, and the remaining 6.6% reside in the allowed region. The overall validation criterion represented an exemplary structure of the predicted GABA receptor β1 model and was suitable for further studies.

The functional GABA receptor 3D structures are reportedly observed in parallel chains, i.e., tail-to-tail and head-to-head conformation^39,55,56^. Hence, to form a functional GABA receptor α1-β1 complex, the retrieved GABA receptor α1 subunit was docked against the modelled GABA receptor β1 using Hex tool 8.0.0^46^. The functional conformation of GABA receptor α1-β1 with parallel binding (head-to-head and tail-to-tail) was selected for further study, with a binding energy of -1206.05 KJ/mol. The structural alignment of the selected GABA receptor α1-β1 docked complex against the reported tertiary structure of the GABA receptor α1-β3 (PDB ID: 6HUJ) yielded an RMSD of 0.099, indicating a high accuracy of the modelled structure. The selected structure of the GABA receptor α1-β1 was subjected to energy minimisation using UCSF Chimera for further docking studies.

### Molecular docking study

The selected 349 drug molecules were energy-minimised and docked against the target proteins for epilepsy, including Nav1.2, GABA receptor α1-β1, and Cav3.1, using AutoDock v4.2.6. Top-selling AEDs, Carbamazepine, Clonazepam, and Pregabalin, were chosen as standard drugs for the studied target receptors - Nav1.2, GABA receptor α1-β1, and Cav3.1, respectively, for comparing the binding affinities of the docked drug complexes^16,50^. The minimum binding energies of standard medications, Carbamazepine, Clonazepam and Pregabalin, were observed to be -7.13 Kcal/mol, -6.14 Kcal/mol and − 5.76 Kcal/mol when docked against protein receptors Nav1.2, GABA receptor α1-β1, and Cav3.1, respectively.

**Fig. 4:**
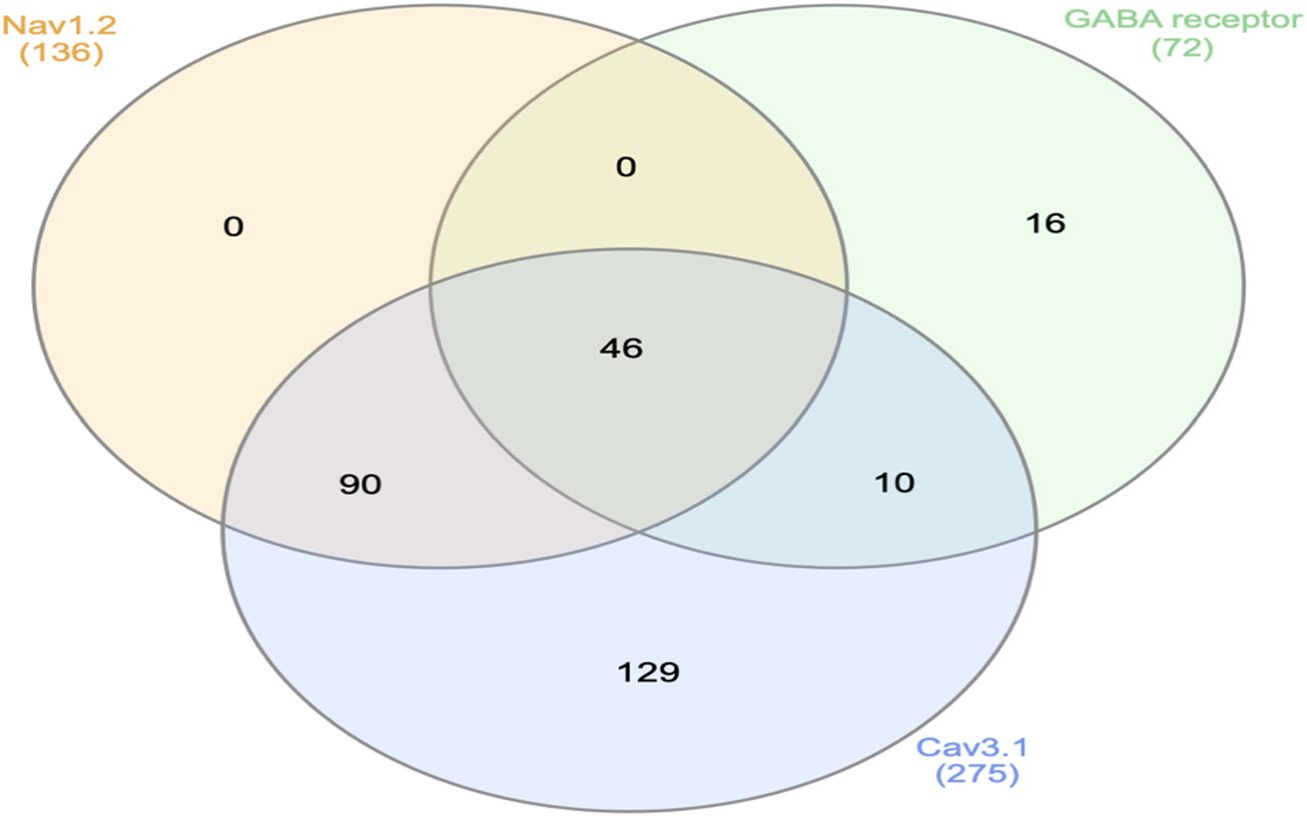
Drugs having better binding affinity as compared to standard drugs for epilepsy receptors, Voltage-gated sodium channel α2 (Nav1.2), GABA receptor α1-β1, and Voltage-gated calcium channel α1G (Cav3.1).

A total of 136 drugs showed better binding affinities for the epilepsy receptor Nav1.2 compared to the standard Carbamazepine, while 72 drugs showed lower binding energies for GABA receptor α1-β1 when compared to GABA agonist Clonazepam. A total of 275 drugs, screened against Cav3.1, showed better binding affinities than the corresponding standard drug, Pregabalin (Supplementary File: S5). These 275 Cav3.1 binding drugs included all 136 Nav1.2 binding drugs and 56 out of 72 GABA receptor α1-β1 binding drugs. Overall, 46 drugs, in common, showed better binding affinities against their corresponding therapeutic target receptors (Fig. 4; Table 4). These 46 drug compounds were also predicted to be BBB permeable and showed structural similarity with already marketed AEDs; thus, they can be expected as potential candidates for AED repositioning.

**Table 4:** Drugs having better binding energies (Kcal/mol) than standard drugs against selected epileptic receptors: Voltage-gated sodium channel α2 (Nav1.2), GABA receptor α1-β1, and Voltage-gated calcium channel α1G (Cav3.1).

DrugBank ID	Name	Nav1.2	GABA receptor α1-β1	Cav3.1
DB00564	Carbamazepine	-7.13	--	--
DB01068	Clonazepam	--	-6.14	--
DB00230	Pregabalin	--	--	-5.76
DB00192	Indecainide	-7.84	-6.45	-9.73
DB00321	Amitriptyline	-7.94	-6.53	-9.56
DB00340	Metixene	-8.53	-6.65	-9.61
DB00344	Protriptyline	-7.68	-6.38	-8.99
DB00427	Triprolidine	-7.89	-6.16	-9.70
DB00434	Cyproheptadine	-8.23	-6.63	-8.46
DB00486	Nabilone	-8.60	-6.50	-8.28
DB00496	Darifenacin	-9.83	-7.22	-9.41
DB00540	Nortriptyline	-8.01	-6.84	-10.16
DB00561	Doxapram	-8.53	-6.63	-8.73
DB00568	Cinnarizine	-9.12	-6.48	-9.44
DB00693	Fluorescein	-7.70	-6.41	-8.01
DB00719	Azatadine	-7.81	-6.47	-8.58
DB00850	Perphenazine	-8.12	-6.25	-10.45
DB00920	Ketotifen	-7.76	-6.67	-8.70
DB00924	Cyclobenzaprine	-8.16	-6.84	-9.29
DB00933	Mesoridazine	-9.12	-6.35	-9.38
DB00934	Maprotiline	-8.15	-7.06	-9.97
DB00972	Azelastine	-9.12	-6.62	-10.26
DB00991	Oxaprozin	-7.61	-6.60	-8.11
DB01009	Ketoprofen	-7.38	-7.26	-7.26
DB01100	Pimozide	-9.11	-6.80	-10.22
DB01142	Doxepin	-7.69	-6.37	-9.46
DB01146	Diphenylpyraline	-8.07	-6.32	-8.96
DB01148	Flavoxate	-8.01	-6.49	-9.08
DB01501	Difenoxin	-8.70	-7.33	-9.46
DB01544	Flunitrazepam	-7.23	-6.27	-7.64
DB01608	Periciazine	-8.30	-6.52	-9.13
DB01623	Thiothixene	-8.83	-6.25	-11.47
DB01624	Zuclopenthixol	-8.17	-6.33	-11.30
DB01628	Etoricoxib	-7.92	-6.63	-7.68
DB04841	Flunarizine	-9.18	-6.21	-9.28
DB05015	Belinostat	-7.41	-7.02	-8.07
DB06077	Lumateperone	-8.84	-7.08	-8.73
DB06153	Pizotifen	-7.69	-6.57	-8.39
DB06413	Armodafinil	-7.19	-6.23	-6.96
DB06626	Axitinib	-8.51	-6.40	-9.38
DB06684	Vilazodone	-9.59	-6.16	-10.93
DB09167	Dosulepin	-7.96	-6.37	-9.42
DB09488	Acrivastine	-7.72	-7.14	-10.23
DB11952	Duvelisib	-9.38	-6.34	-8.41
DB12492	Piritramide	-9.50	-7.08	-11.46
DB12877	Oxatomide	-9.43	-6.43	-9.10
DB13292	Pimethixene	-8.36	-6.96	-8.24
DB14033	Acetyl sulfisoxazole	-7.58	-6.52	-6.50
DB12792	Boscalid	-7.37	-6.14	-7.72

### Binding pocket analysis of selected drugs

The 46 drugs selected above were further evaluated for their binding interactions within the binding pockets of the target proteins. Most marketed AEDs working as VGSC inhibitors bind within the inner pore-loop (P-loop) region. The standard drug Carbamazepine forms a hydrogen bond (H-bond) with the Leu939 residue located within the P-loop region of Domain II. Amino acids Ser1461, Phe1462, and Ile1457 within the P-loop region of VGSC-Domain-III were reported to be essential for the binding of VGSC-inhibiting AEDs to generate an anti-epileptic activity^57–59^. 20 out of 46 selected drugs showed their binding within the binding pocket, including the P-loop region. Drugs Belinostat, Dosulepin, Flavoxate, Mesoridazine, and Oxatomide were observed to form H-bonds within the P-loop region of Domain-II. Drugs Amitriptyline, Azatadine, Azelastine, Cyproheptadine, Difenoxin, Maprotiline, Nortriptyline, Oxaprozin, and Piritramide were observed to form H-bonds within the P-loop region of Domain-III (Supplementary File: S6). Among them, Dosulepin and Flavoxate were observed to form H-bonds with Leu939, while drugs Azatadine, Azelastine, Cyproheptadine and Oxaprozin were forming H-bonds with the key amino acid Ser1461 within the P-loop region of VGSC Domain-III. Drugs Cinnarizine, Darifenacin, Flunarizine, Lumateperone, Pimozide, and Pizotifen did not show any bonded interaction but bound within the P-loop region through non-bonded interactions, while the remaining 26 drugs were not observed to bind within the binding pocket as standard drugs.

GABA and its agonist bind between the α1 and β1 chains of the GABA receptor within the aromatic box, hence creating a hydrophobic environment suitable for neurotransmitter binding. The aromatic region includes Tyrosine (β-Tyr97, β-Tyr157, β-Tyr205) and Phenylalanine (α-Phe64, α-Phe65, and β-Phe200) residues, along with Arginine (α-Arg66, α-Arg67, α-Arg131, α-Arg132, and β-Arg207), which are involved in Electrostatic interactions. Polar amino acids like Threonine (α-Thr129 and β-Thr202) and Serine (α-Ser168) form a Hydrogen bond, while Glutamic acid (β-Glu155 and β-Glu220) contributes to binding affinity by forming salt bridges^60–62^. The cryo-EM structures of Benzodiazepine interaction with the α1β1γ2S tri-heteromeric GABA receptor show ligand interactions with residues in the β-subunit’s Loop A/B/C (Tyr97, Glu155, Tyr157, Tyr205) and make other non-bonded interactions with nearby amino acids, such as Thr129, Thr202, and Arg66^63^. The selected standard drug, Clonazepam, exhibited a polar interaction with α1-Asp184, which is structurally consistent with the α-face contribution to the orthosteric pocket observed in the cryo-EM models. Along with this, several non-bonded interactions with β1-Leu124, β1-Asn125 (adjacent to β6 Thr129), β1-Met162 (neighbouring Tyr157 in Loop B), and β1-Glu178, β1-Arg187 (nearby Thr202 in Loop-C arc) were observed. These are pocket-lining positions flanking the canonical determinants and are well-placed to form auxiliary van der Waals or polar contacts. While these residues are not the classic β-face ‘signature’ triad, their proximity to Loop A–C and β2/β6 strands provides a structurally sound rationale for the observed interactions. Among selected drugs, 16 drugs (Acetyl sulfisoxazole, Acrivastine, Armodafinil, Axitinib, Azatadine, Cyclobenzaprine, Cyproheptadine, Etoricoxib, Flunitrazepam, Ketotifen, Maprotiline, Nortriptyline, Oxaprozin, Pizotifen, Protriptyline, and Vilazodone) showed their binding within the binding region of GABA receptor subunit α1 with the formation of an H-bond. Among these, Acrivastine, Doxepin, Duvelisib, Flunitrazepam, Oxaprozin, and Pizotifen showed H-bonding with Asp184 of the GABA receptor α1 subunit. Drugs Acetyl sulfisoxazole, Armodafinil, Cyclobenzaprine, Maprotiline, Nortriptyline, and Protriptyline were observed to form an H-bond with Asp63 of subunit α1. Drugs Azatadine, Belinostat, Cyproheptadine, Difenoxin, Ketotifen, Lumateperone, Nabilone, and Pizotifen were observed to form H-bonds with the amino acids Leu124, Asn125, Lys127, Met162, Asn174, and Thr176 from the GABA receptor subunit β1. In contrast, Pimethixene and Triprolidine did not show any H-bonding but were observed to bind within the binding pocket of GABA receptor α1-β1 (Supplementary File: S6).

The pore-forming intracellular region of S5 and S6 in VGCC has been reportedly to be necessary for the trafficking of Ca^+ 2^ ions^64^; hence, drug binding within this region would generate a therapeutic response for epileptic seizures. The standard drug, Pregabalin, showed three H-bonded interactions with amino acids Glu354 (2.72Å), Gln922 (2.77Å), and Glu923 (2.7Å) within the S6 helical region of repeats I and II. All the selected drugs were observed to bind within the binding pocket of Cav3.1, similar to the standard drug. However, the H-bond analysis, in comparison to the standard drug Pregabalin, revealed that 25 drugs share common amino acids. Among those, Amitriptyline, Dosulepin, Doxepin, Flavoxate, Maprotiline, Metixene, Nortriptyline, Pizotifen and Protriptyline showed H-bond with Glu354; Boscalid, Doxapram, Etoricoxib, Fluorescein, Ketotifen, Nabilone, Oxaprozin, Piritramide and Thiothixene showed H-bond with Gln922, while drugs Indecainide and Pimozide were observed to form H-bond with Glu923. Drugs Acrivastine, Cyproheptadine, and Ketotifen formed hydrogen bonds with Glu354 and Gln922. In comparison, Cyclobenzaprine and Triprolidine were observed to form H-bonds with Glu354 and Glu923 (Supplementary File: S6).

The binding pocket comparison of selected drugs revealed six drugs (Azatadine, Cyproheptadine, Maprotiline, Nortriptyline, Oxaprozin, and Pizotifen) with binding pockets similar to those of standard drugs for their respective target receptors. The binding pocket analysis of these drugs confirms their ability to mimic the pharmacodynamics of the standard drugs. These six drug hits were further evaluated for their role in seizure management, using exhaustive text mining.

### Literature data mining

The selected marketed drugs (Azatadine, Cyproheptadine, Maprotiline, Nortriptyline, Oxaprozin, and Pizotifen) belong to different classes of drugs and were analysed for their previous reports in the context of epileptic seizures and other toxicity assays.

### Azatadine

Azatadine is a first-generation antihistamine that primarily functions as an H1 receptor antagonist, demonstrating efficacy in treating allergic reactions such as rhinitis, conjunctivitis, and urticaria^65^. Its chemical structure is closely related to tricyclic compounds, contributing to its ability to cross the blood-brain barrier. As a result, Azatadine can cause significant CNS effects, such as drowsiness and sedation^66^. The sedative effects of Azatadine may lower the seizure threshold, potentially increasing the risk of seizures, especially in individuals with a history of epilepsy or other neurological conditions. Additionally, its anticholinergic effects, such as dry mouth, blurred vision, and urinary retention, may interfere with the normal neurotransmitter balance in the brain, thereby complicating seizure management and other neurological functions^67^. Azatadine is usually taken orally and may interact with other CNS depressants like alcohol, sedatives, or tranquillisers, enhancing its sedative and central effects. Considering these factors, particularly in patients with seizure disorders, Azatadine is generally contraindicated and has been excluded from our study outcomes to avoid confounding effects related to neurological safety.

### Cyproheptadine

Cyproheptadine is also classified as a first-generation antihistamine with anticholinergic, antiserotonergic, and sedative properties. It was initially developed to treat allergies by blocking the H1 histamine receptors; furthermore, it is also widely used to stimulate appetite, especially in patients experiencing cachexia or malnutrition^68,69^. It is also prescribed for the treatment of serotonin syndrome, a potentially life-threatening condition caused by excessive serotonergic activity in the CNS. The serotonin receptors, particularly the 5-HT2A receptor, are implicated in seizure activity. In some cases of intractable epilepsy, where patients do not respond well to conventional anticonvulsants, Cyproheptadine has been used off-label to help control seizures by modulation of serotonergic pathways, which can influence neuronal excitability and seizure thresholds. Animal studies have also shown a reduction in seizure frequency and neural cell death following Cyproheptadine treatment^70^. Additionally, the drug’s antiserotonergic effects may influence other serotonergic-related conditions, including migraine prevention and specific psychiatric disorders. Its sedative properties are also utilised in managing symptoms of anxiety and sleep disturbances.

### Maprotiline

Maprotiline is a tetracyclic antidepressant (TeCA) primarily used to treat depression, particularly major depressive disorder and dysthymia. It functions by inhibiting the reuptake of norepinephrine, a neurotransmitter, thereby increasing its levels in the brain and alleviating depressive symptoms^71,72^. Unlike tricyclic antidepressants (TCAs), which affect multiple neurotransmitters, Maprotiline is more selective for norepinephrine, which reduces some of the side effects typically associated with TCAs. Additionally, it has strong sedative properties, which makes it worthwhile for patients with anxiety or insomnia, but potentially problematic for those who are sensitive to sedatives^71^. Maprotiline has also been explored for its potential effects on epilepsy, but it is not a first-line treatment. The clinical studies suggest that Maprotiline can lower the seizure threshold; however, at higher doses or in patients with a predisposition to seizures, it behaves as a proconvulsant^73,74^. Therefore, Maprotiline use in epileptic patients is generally approached with caution, often requiring close monitoring and consideration of alternative therapies.

### Nortriptyline

Nortriptyline is another TCA, primarily used to treat major depressive disorder; however, it is also prescribed for conditions such as chronic pain, neuropathic pain, and particular anxiety disorders^75–77^. It works by inhibiting the reuptake of neurotransmitters, particularly norepinephrine and, to a lesser extent, serotonin, in the brain, which enhances the mood-stabilising effects of these chemicals. This enhancement may serve to alleviate depressive symptoms and improve pain management. Furthermore, it demonstrates sedative properties, rendering it advantageous in cases involving anxiety and insomnia. The medication is available in a generic formulation and typically represents a cost-effective alternative to newer antidepressants, although it may be associated with a higher incidence of adverse effects. These adverse effects include dry mouth, blurred vision, dizziness, urinary retention, and constipation. Due to its anticholinergic properties, caution is recommended when prescribing to elderly patients to prevent adverse events such as confusion, falls, and cognitive decline. Nortriptyline is reported to lower the seizure threshold, which can increase the risk of seizures in susceptible individuals^78,79^. Consequently, its use should be carefully considered in patients with a history of seizures or predisposition thereto. Nonetheless, in certain circumstances, it may be appropriate where an epileptic patient suffers from comorbid conditions like depression or chronic pain, where the benefits of its mood-stabilising effects might outweigh the seizure risks. Hence, the use of nortriptyline in patients with epilepsy involves a careful risk-benefit analysis, considering the individual’s seizure history, the severity of comorbid conditions, and the potential for drug interactions.

### Oxaprozin

Oxaprozin is a non-steroidal anti-inflammatory drug (NSAID) commonly used to treat inflammation or pain caused by osteoarthritis or rheumatoid arthritis. It inhibits the cyclooxygenase (COX) enzyme non-selectively, thereby reducing the synthesis of prostaglandins and lipid compounds; consequently, inflammation, pain and fever are reduced^80^. Oxaprozin is distinguished by its prolonged half-life, which allows for once-daily administration, thereby enhancing patient compliance and adherence. It is generally well-tolerated; however, potential adverse effects include gastrointestinal discomfort, cardiovascular complications, and alterations in renal function, particularly with prolonged usage. Although it is widely utilised for inflammatory conditions. However, Oxaprozin has not been widely studied or recommended for use in individuals with epilepsy. Public databases and clinical trials have not documented seizure-related side effects associated with its administration. Notably, recent research indicates a potential new therapeutic application for Oxaprozin. Specifically, an in vivo study involving seizure models in rats demonstrated that Oxaprozin may possess anticonvulsive properties during the behavioural assessment^81^. This finding paves the way for further investigation into its potential use as an adjunctive treatment for epilepsy or seizure disorders, representing a promising frontier for future pharmacological innovation.

### Pizotifen

Pizotifen is a tricyclic drug primarily used as a preventive treatment for migraine headaches and cluster headaches. It functions as a serotonin receptor antagonist, explicitly targeting the 5-HT2A and 5-HT2C receptors, and exhibits antihistamine and anticholinergic properties^82,83^. Inhibiting serotonin, a neurotransmitter believed to play a role in the dilation of blood vessels in the brain, results in reduced frequency and severity of migraines. Pizotifen is often prescribed when other treatments, such as beta-blockers or anti-epileptic drugs, are either ineffective or unsuitable for the patient. Its role in epilepsy is less prominent and more complex, as it has been observed to exhibit some anticonvulsant properties in adult zebrafish by modulating serotonin levels within the brain^84^. However, its efficacy in epilepsy is not well-established, and it is generally not considered a standard anti-epileptic drug. The use of Pizotifen in epilepsy might be explored in patients with comorbid conditions, such as migraines, where the dual action of preventing headaches and potentially reducing seizures could be beneficial. Nonetheless, more research is required to better understand its exact mechanism and effectiveness in managing epilepsy.

Based on their pharmacological profiles and existing evidence, Cyproheptadine, Oxaprozin, and Pizotifen are promising candidates for epilepsy repurposing, with Cyproheptadine’s antiserotonergic effects and prior off-label success in seizure management emphasising its potential. Oxaprozin has demonstrated anticonvulsant activity in animal models, warranting further investigation, while Pizotifen’s antagonism of serotonin receptors suggests therapeutic benefits, particularly for patients experiencing migraines and seizures. Conversely, Azatadine, Maprotiline, and Nortriptyline pose safety concerns, primarily due to their propensity to lower seizure thresholds and induce CNS complications, which leads to their exclusion from future studies. Therefore, we propose Oxaprozin, Pizotifen, and the antihistamine Cyproheptadine as potential multitarget AEDs for various types of epileptic seizures, regardless of their origin—whether genetic, congenital, due to physical injury, or environmental.

#### Molecular dynamics simulation study

The docked complex of selected potential multi-targeted AED candidates—Cyproheptadine, Oxaprozin, and pizotifen—with the epilepsy target receptors Nav1.2, GABA receptor α1-β1, and Cav3.1—was subjected to molecular dynamics studies to evaluate their stability in physiological conditions. All the receptor-drug complexes showed a minimised energy configuration within a 3000 ps run, followed by equilibration at an average temperature and pressure of 310 K and one psi, respectively. The docked complexes of the selected drugs were finally simulated for a 100-ns runtime in triplicate.

### Root mean square deviation (RMSD)

**Fig. 5:**
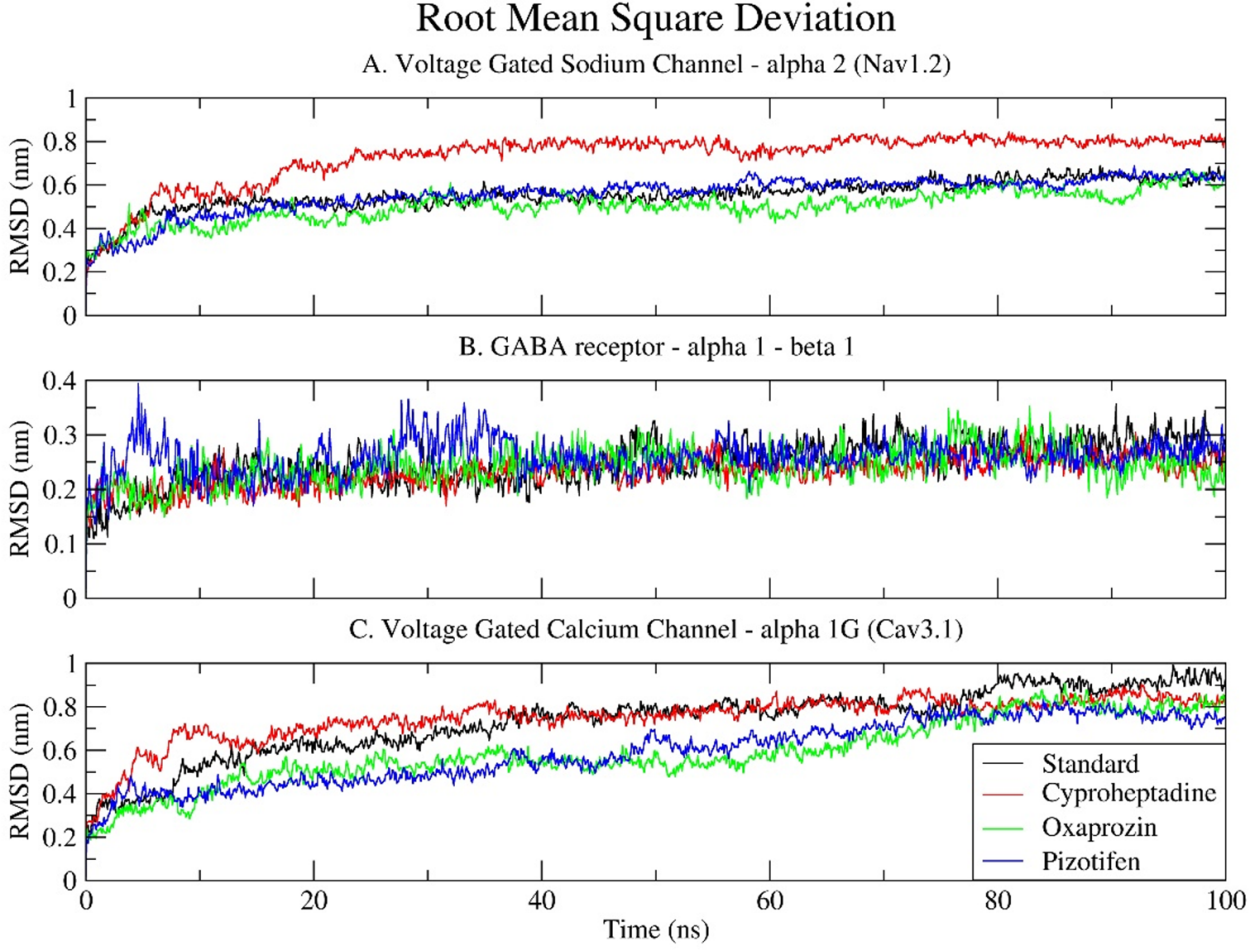
Time series plot depicting the Root Mean Square Deviation (RMSD) between the selected epilepsy proteins: (A) Voltage-gated sodium channel α2 (Nav1.2); (B) GABA receptor α1-β1; and (C) Voltage-gated calcium channel α1G (Cav3.1) and selected drugs (Standard, Cyproheptadine, Oxaprozin, Pizotifen) over a 100 ns simulation.

The RMSD analysis measures the structural stability (average distance between the atoms of superimposed molecules) and conformational shifts of protein-ligand complexes throughout MDS. For all simulated complexes, the RMSD graph appeared to stabilise within the initial 10-ns run and remained stable until the end of the simulation (Fig. 5).

Regarding the Nav1.2 sodium channel, all ligand-bound complexes demonstrated an upward trend in RMSD during the initial 20–30 ns, indicative of structural adjustments and system equilibration. The standard drug, Carbamazepine, forms a comparatively stable complex, with RMSD values stabilising around 0.5 to 0.6 nm following an initial equilibration phase. Pizotifen and Oxaprozin exhibit marginally lower deviations, stabilising within the range of 0.45 to 0.55 nm, thereby suggesting an enhancement in structural stability relative to the reference. Conversely, Cyproheptadine results in significantly higher deviations, with RMSD values gradually rising to approximately 0.7 to 0.8 nm. This implies that Cyproheptadine may induce destabilisation of Nav1.2 or facilitate increased conformational flexibility, potentially attributable to suboptimal binding interactions or alterations in key structural regions. Despite the RMSD reaching a plateau, the consistently elevated values associated with Cyproheptadine suggest a less stable complex formation compared to the other ligands (Fig. 5A).

The GABA receptor α1-β1 exhibits notably stable behaviour across all ligand-bound forms. Throughout the 100 ns simulation, the RMSD values for the four ligand systems remain consistently low, typically ranging from 0.15 to 0.3 nm. This minimal variation indicates a high degree of structural rigidity, suggesting that the GABA receptor maintains its native conformation irrespective of the ligand bound. The RMSD trajectories of the standard drugs, Oxaprozin, Cyproheptadine, and Pizotifen, nearly coincide, with only minor and transient deviations, particularly with Pizotifen. This observation implies that the binding of any of these compounds does not substantially modify the receptor’s backbone architecture, thereby underscoring the receptor’s intrinsic stability and robustness (Fig. 5B). The lower RMSD in this case may also be attributed to the smaller size of the receptor complex and the limited flexibility of its core structural regions.

Regarding Cav3.1, the RMSD patterns bear a close resemblance to those observed in Nav1.2. The standard drug, Pregabalin, maintains the receptor stability within a range of 0.65 to 0.75 nm. Pizotifen and Oxaprozin demonstrate marginally lower deviations, sustaining RMSD values of approximately 0.55–0.65 nm, thereby implying that they facilitate stable receptor-ligand interactions. Cyproheptadine exhibits the most considerable deviation, approaching 0.85 nm, which suggests it may induce greater conformational alterations or flexibility within the calcium channel. Although all examined systems appear to attain equilibrium after roughly 40 nanoseconds, the elevated RMSD associated with Cyproheptadine indicates a comparatively less stable interaction, potentially affecting the channel’s structural integrity or functionality (Fig. 5C).

The relatively high RMSD values observed in the Nav1.2 and Cav3.1 complexes, particularly with Cyproheptadine, do not necessarily indicate instability. Instead, they are likely attributable to the substantial size and intricate structure of these transmembrane ion channels, which encompass multiple domains and flexible loops. These proteins inherently exhibit greater conformational flexibility, especially in extracellular and intracellular loops or unresolved terminal regions that may have been modelled during structure preparation. RMSD values ranging from 0.6 to 0.8 nm are typical in such large membrane proteins and are deemed acceptable, particularly when the RMSD plateaus, indicating that the system has achieved equilibrium without substantial structural compromise.

In summary, all three receptor–ligand systems reach equilibrium during the simulation. Oxaprozin and Pizotifen generally maintain or enhance structural stability compared to standard drugs. Although Cyproheptadine causes higher RMSD in both voltage-gated ion channels, these values remain within acceptable limits, indicating dynamic rearrangements rather than destabilisation. The GABA receptor complexes, being smaller and more rigid, show minimal deviation across all ligands, demonstrating strong stability. Overall, these findings suggest that Oxaprozin and Pizotifen are more structurally advantageous binders and could offer improved therapeutic stability.

### Root mean square fluctuation (RMSF)

**Fig. 6:**
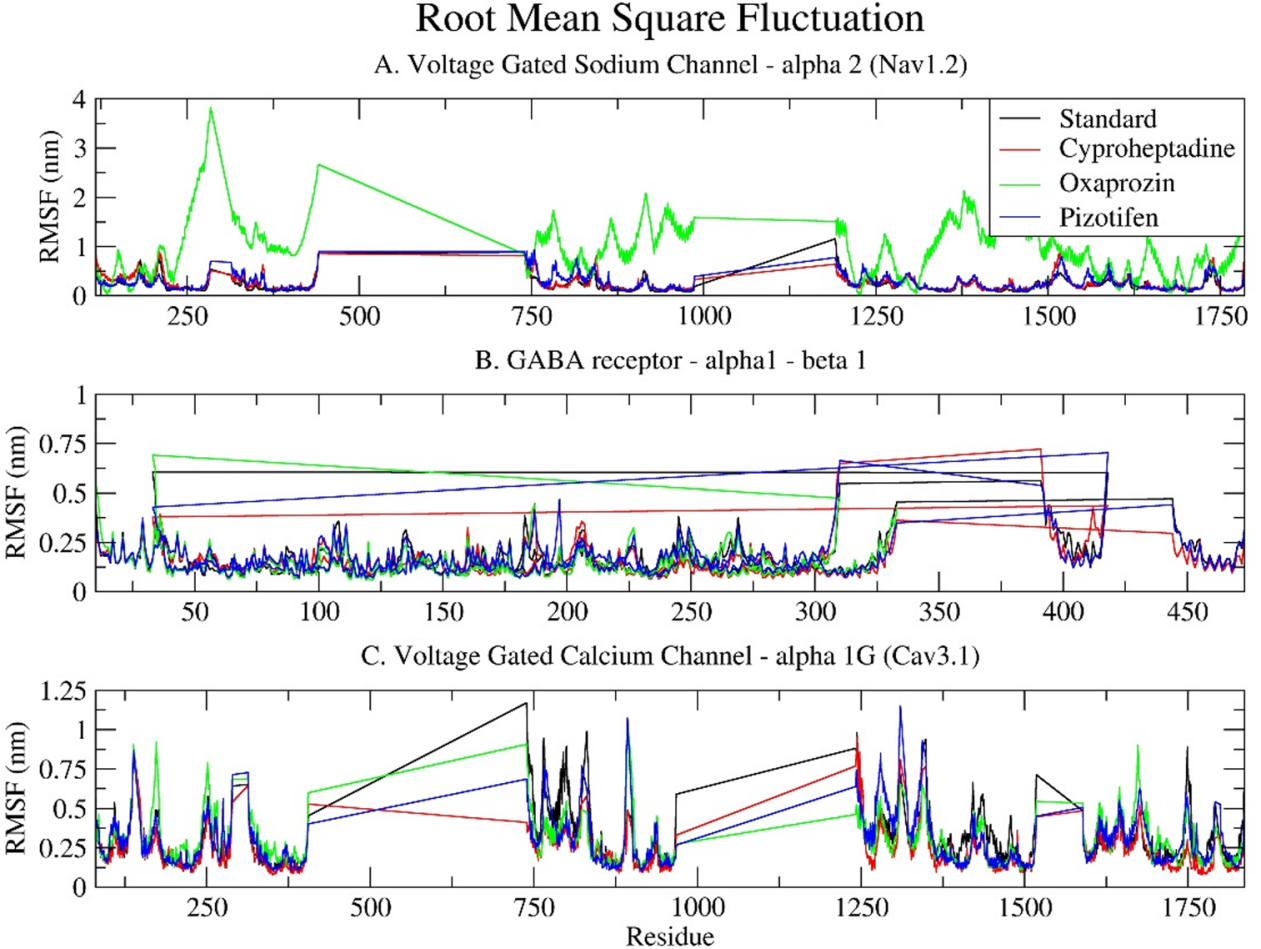
Time series plot depicting the Root Mean Square Fluctuation (RMSF) between the selected epilepsy proteins: (**A**) Voltage-gated sodium channel α2 (Nav1.2); (**B**) GABA receptor α1-β1; and (**C**) Voltage-gated calcium channel α1G (Cav3.1) and selected drugs (Standard, Cyproheptadine, Oxaprozin, Pizotifen) over a 100 ns simulation.

The RMSF measures the average deviation, flexibility, and dynamic behaviour of a particle (e.g., protein residue) over time from a reference position (typically the time-averaged position of the particle) during molecular dynamics (MD) simulations. It analyses the portions of the structure that fluctuate from their mean structure the most or least. The Cyproheptadine drug showed the lowest RMSF, followed by Pizotifen and the standard drugs, while Oxaprozin exhibited the maximum RMS fluctuation upon binding to all three selected epilepsy target receptors (Fig. 6).

Regarding Nav1.2, the standard drug Carbamazepine exhibits low to moderate fluctuations along the polypeptide chain, indicative of a stable receptor-ligand interaction. Cyproheptadine and Pizotifen demonstrate RMSF profiles analogous to the standard, suggesting their binding does not significantly alter the conformational stability of Nav1.2. Conversely, Oxaprozin induces notable fluctuations in specific regions, particularly residues 150–300, 700–900, and 1000–1300, with RMSF values reaching up to 3.16 nm, which might be due to bonding and non-bonding interactions. These fluctuations suggest increased local flexibility or destabilisation, which could potentially affect the gating function of the sodium channel. Furthermore, the RMSF plot displays flat, horizontal lines between the fluctuating regions, representing unresolved residues in the experimental model and are thus excluded from the molecular dynamics simulations. These lines are visual artefacts indicating missing segments, rather than actual data (Fig. 6A).

The standard drug Clonazepam exhibits relatively low RMSF values for the GABA receptor α1-β1, with most values being below 0.5 nm, indicating a stable complex. Cyproheptadine and Pizotifen display slightly higher flexibility in certain regions, particularly around residues 280–320 and 380–420, likely corresponding to extracellular or cytoplasmic loops. Oxaprozin exhibits a slight fluctuation near residue 50 but generally resembles the standard closely. This suggests all three drugs maintain the structural integrity of the GABA receptor, with only localised, non-disruptive flexibility variations. The RMSF plot distinctly shows the α1 and β1 regions, with separate plots and no connecting lines, highlighting their physical separation. (Fig. 6B).

In the Voltage Gated Calcium Channel alpha 1G (Cav3.1), all four ligands, including the Standard, exhibit highly similar fluctuation patterns, with minor peaks near residues 800 and 1400. Cyproheptadine and Pizotifen closely follow the Standard, suggesting they have little effect on the receptor’s overall flexibility. Conversely, Oxaprozin shows slightly increased fluctuations across several regions, especially between residues 200–400, 750–900, and 1200–1500, corresponding to Domains I and IV. Though less severe than in Nav1.2, these fluctuations suggest moderate destabilisation. As with the sodium channel, the RMSF plot for Cav3.1 displays straight lines, indicating gaps in the resolved structural data (see Fig. 6C).

Compared to the standard drug, Cyproheptadine and Pizotifen exhibit similar structural behaviours across all three receptors, as evidenced by their comparable RMSF profiles, which denote the preservation of native conformational dynamics. This correlates with their moderate RMSD values, indicating stable overall structures with only localised fluctuations. Conversely, Oxaprozin displays a distinct pattern characterised by increased residue-level flexibility, particularly in the loop and inter-domain regions of Nav1.2 and Cav3.1 channels. This heightened flexibility is demonstrated by its higher RMSD values, suggesting potential ligand-induced conformational changes or allosteric effects. Such deviations may indicate a different binding mechanism, which could subsequently influence receptor modulation.

### Radius of gyration (Rg)

**Fig. 7:**
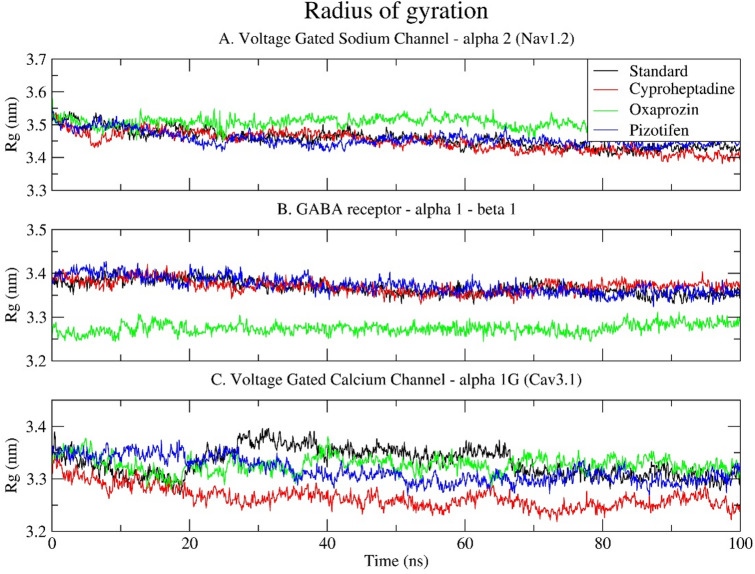
Time series plot depicting the Radius of Gyration (Rg) between the selected epilepsy proteins: (**A**) Voltage-gated sodium channel α2 (Nav1.2); (**B**) GABA receptor α1-β1; and (**C**) Voltage-gated calcium channel α1G (Cav3.1) and selected drugs (Standard, Cyproheptadine, Oxaprozin, Pizotifen) over a 100 ns simulation.

Rg measures the distribution of protein atoms around its centre of mass, giving key insights into the overall compactness and stability of receptor-ligand complexes during simulation. All three target receptors exhibited stable atomic distributions, ranging from 3.2 to 3.6 nm (Fig. 7). For Nav1.2, complexes maintained consistent Rg values over 100 ns, indicating no significant unfolding (Fig. 7A). The Oxaprozin-bound complex had slightly higher Rg values than the standard and other ligands, hinting at a mild expansion of the protein structure, which aligns with its higher RMSD and RMSF. In the GABA receptor α1–β1 complex, the standard, Cyproheptadine, and Pizotifen complexes exhibited similar Rg profiles, ranging from 3.35 to 3.4 nm, indicating consistent compactness. Oxaprozin showed a lower Rg (~ 3.25–3.3 nm), possibly reflecting tighter packing or structural constriction from its binding mode (Fig. 7B). In the calcium channel (Cav3.1), standard and Pizotifen complexes maintained stable, compact Rg trajectories. Cyproheptadine’s Rg slightly decreased over time, indicating increased compactness, while Oxaprozin’s Rg stayed intermediate but stable. These results align with earlier RMSD and RMSF data, where Cyproheptadine and Pizotifen had moderate dynamics, and Oxaprozin showed more variation depending on the receptor (Fig. 7C). Overall, Rg analysis supports the RMSD and RMSF findings, suggesting that Oxaprozin may influence receptor conformations by causing slight expansion (Nav1.2) or compaction (GABA receptor), while Cyproheptadine and Pizotifen tend to maintain structures similar to those of the standard ligand.

### Solvent accessible surface area (SASA)

**Fig. 8:**
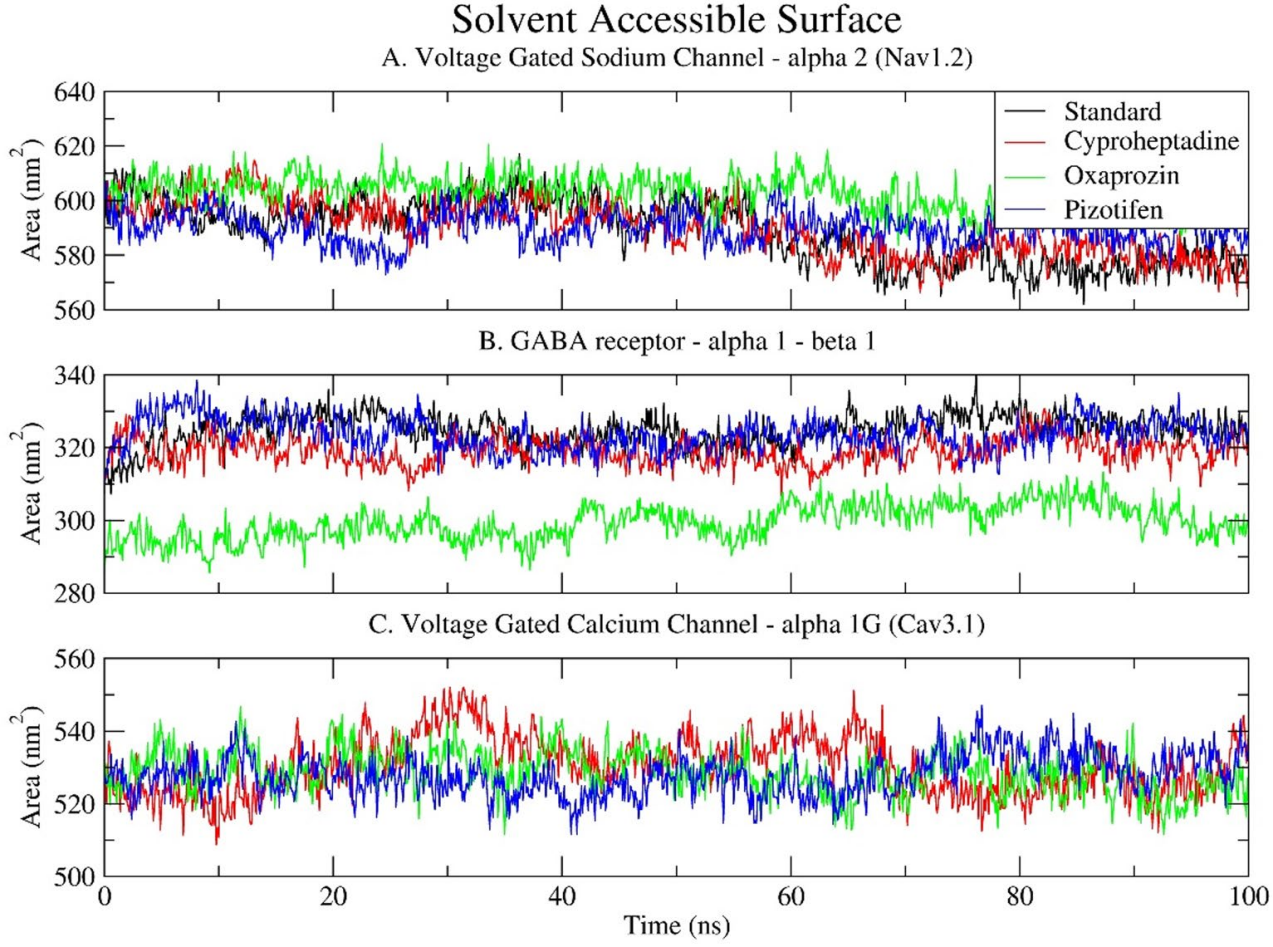
Time series plot depicting the Solvent Accessible Surface Area (SASA) between the selected epilepsy proteins: (**A**) Voltage-gated sodium channel α2 (Nav1.2); (**B**) GABA receptor α1-β1; and (**C**) Voltage-gated calcium channel α1G (Cav3.1) and selected drugs (Standard, Cyproheptadine, Oxaprozin, Pizotifen) over a 100 ns simulation.

SASA is a critical parameter that describes the area surrounding a macromolecule accessible to solvent molecules. It aids in understanding protein folding, ligand binding, and the conformational flexibility and solvent exposure of protein-ligand complexes, which are fundamental for examining molecular interactions at the atomic level. For all drug-receptor complexes, the SASA graphs remained stable, indicating the overall size of the protein (see Fig. 8). The higher SASA values for Nav1.2 (580 nm² – 620 nm²) and Cav3.1 (510 nm² – 550 nm²) in comparison to the GABA receptor α1-β1 (290 nm² – 340 nm²) reflect their larger structures, with Nav1.2 and Cav3.1 containing more than 1000 amino acids, whereas the GABA receptor α1-β1 comprises 602 amino acids.

In Nav1.2, all ligand-bound complexes exhibit relatively stable SASA values, ranging from approximately 590 to 610 nm². Oxaprozin, however, demonstrates a marginally higher SASA (608 nm²) in comparison to the typical 590 nm² observed for Cyproheptadine and Pizotifen. This indicates a more expanded or loosely packed structure, consistent with its elevated Rg and RMSF values, which signify increased flexibility and conformational diversity (Fig. 8A).

In the GABA receptor α1–β1, SASA trends are more noticeable. Oxaprozin exhibits a notable decrease in solvent accessibility, averaging about 302 nm², in contrast to the standard and other drugs, which range from 320 to 330 nm². This suggests a more compact protein conformation, consistent with the lower Rg observed in this complex, indicating possible structural tightening or different folding when Oxaprozin binds. This also highlights Oxaprozin’s unique interaction pattern with GABA receptor residues (see Fig. 8B).

All Cav3.1 complexes show moderate SASA fluctuations between 520 and 540 nm², with no significant changes. Cyproheptadine and Pizotifen have slightly higher and more stable SASA values compared to Oxaprozin, indicating better solvent exposure while maintaining stability, which is also reflected in their consistent Rg and RMSD measures. Conversely, Oxaprozin displays marginally lower SASA, suggesting more internalised or compact areas, possibly due to changes in binding-induced topology (Fig. 8C).

Along with the Rg and RMSD data, the SASA analysis confirms that Cyproheptadine and Pizotifen preserve solvent-exposed, compact, and stable receptor conformations. Conversely, Oxaprozin induces receptor-specific conformational modifications, either expansion in Nav1.2 or contraction in the GABA receptor, that may influence ligand binding and receptor functionality.

Principal Component Analysis (PCA).

**Fig. 9:**
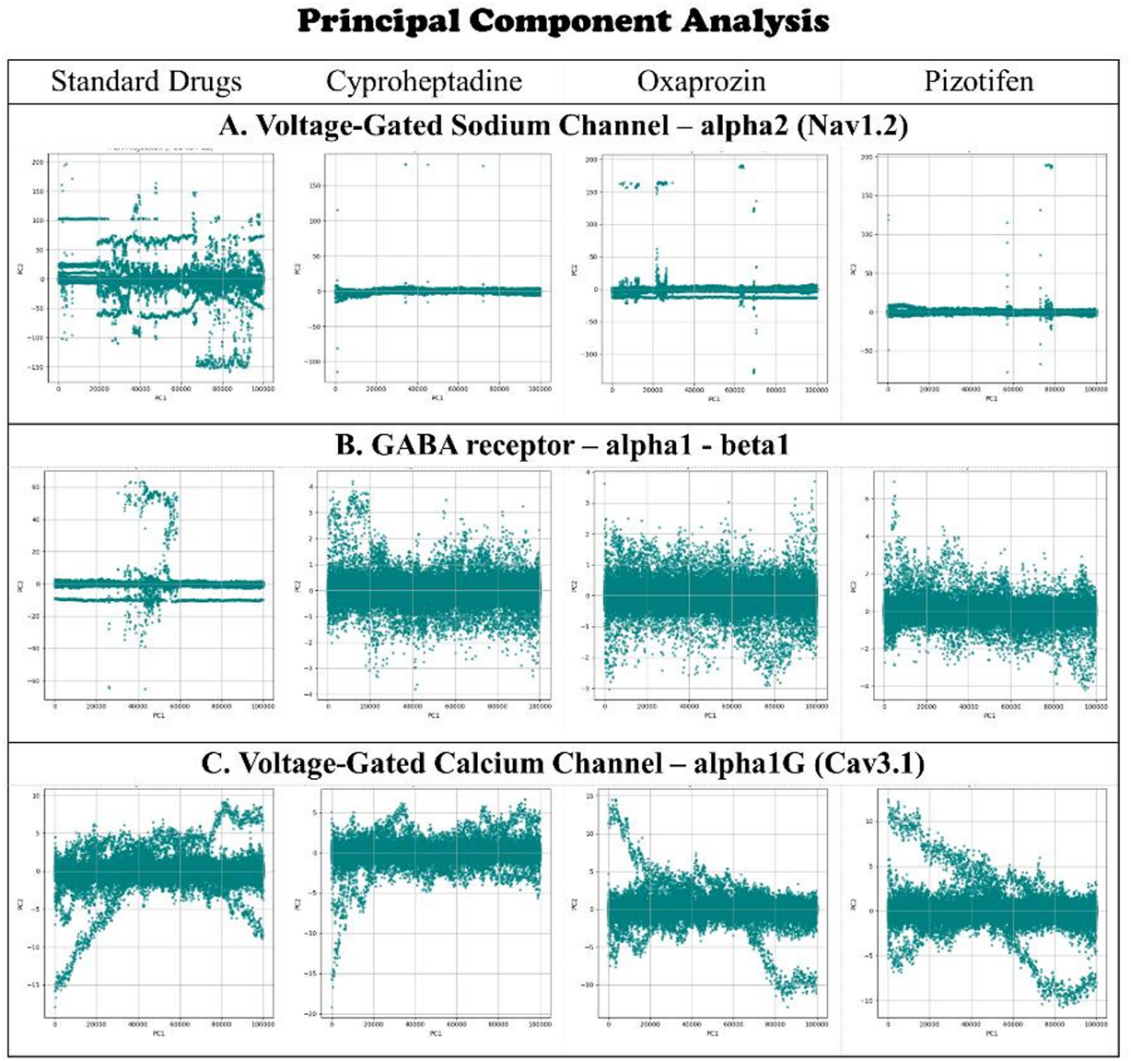
PCA plots illustrating the conformational variability of protein-ligand complexes for selected epilepsy proteins: (**A**) Voltage-gated sodium channel α2 (Nav1.2); (**B**) GABA receptor α1-β1; and (**C**) Voltage-gated calcium channel α1G (Cav3.1) and selected drugs (Standard, Cyproheptadine, Oxaprozin, Pizotifen) over a 100 ns simulation.

PCA was employed to analyse the overall motions and essential dynamics of the protein-ligand complexes throughout the simulation. This technique reduces complex atomic movements into principal components (PCs), with the initial few PCs accounting for the majority of conformational variability. In the case of Nav1.2, the standard drug Carbamazepine, alongside Cyproheptadine and Pizotifen, cluster closely within the PC1 versus PC2 space, indicating a limited conformational range and suggesting stabilised dynamics with minimal large-scale structural alterations. Conversely, Oxaprozin occupies a broader, more dispersed region across the PCs, signifying greater structural flexibility and dynamic variability (Fig. 9A). This observation aligns with elevated RMSD, Rg, and SASA values for the same receptor, thereby reinforcing the conclusion that Oxaprozin binding results in a more flexible or destabilised conformation.

The PCA plot for the GABA receptor α1-β1 shows tighter clustering for Cyproheptadine and Pizotifen, suggesting limited movement and stable complex formation. Oxaprozin also exhibits restricted mobility in PCA space, aligned with its lower SASA and Rg values, which indicate a more compact or rigid structure. This suggests that Oxaprozin binds in a manner that restricts the motion of key domains, potentially impacting its functional dynamics (Fig. 9B). In Cav3.1, PCA patterns display moderate clustering across all ligands, with Cyproheptadine and Pizotifen forming slightly more cohesive groups than Oxaprozin, hinting at more uniform dynamics. However, these differences are less marked than in Nav1.2 or the GABA receptor, suggesting Oxaprozin does not significantly destabilise Cav3.1 but does influence its motion more than the standard drug and other compounds (Fig. 9C). Overall, PCA provides a clear view of the complexes’ dynamical behaviour, showing Cyproheptadine and Pizotifen promote stable, functionally consistent dynamics across all three receptors. In contrast, Oxaprozin causes receptor-specific changes in key motions by either increasing flexibility or rigidity, thereby setting its interaction profile apart from that of the standard drug.

### Interaction energy

**Fig. 10:**
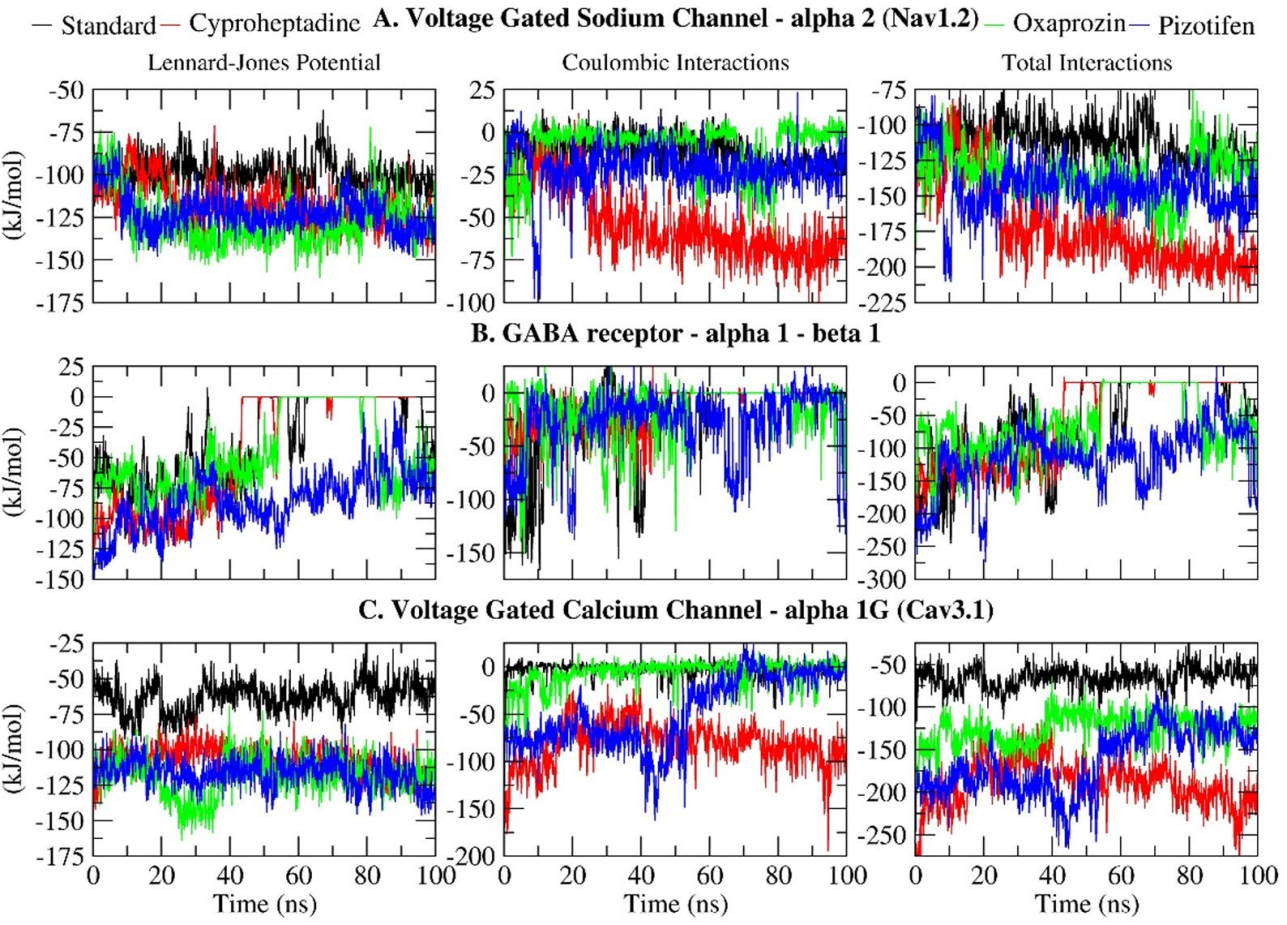
Time series plot depicting the interaction energy (Lennard-Jones Potential, Coulombic Interactions and Total interactions) between the selected epilepsy proteins: (**A**) Voltage-gated sodium channel α2 (Nav1.2); (**B**) GABA receptor α1-β1; and (**C**) Voltage-gated calcium channel α1G (Cav3.1) and selected drugs (Standard, Cyproheptadine, Oxaprozin, Pizotifen) over a 100 ns simulation.

The interaction energy analysis provides valuable insights into the non-bonding forces that influence the stability of the ligand–receptor complex during Molecular Dynamics Simulation (MDS). This analysis evaluates Lennard-Jones (van der Waals) and Coulombic (electrostatic) interactions, enabling an assessment of the ligand’s binding affinity and potency within the active site. In all three receptor complexes (Nav1.2, GABA receptor α1–β1, and Cav3.1), selected drugs demonstrated stronger interaction energies than their respective standard drugs, indicating potentially more stable and favourable binding (Fig. 10; Table 5).

**Table 5:** Interaction energy (KJ/mol) between selected drugs and epilepsy receptor Nav1.2, GABA receptor alpha1-beta1 and Cav3.1.

	Lennard-Jones Potential (KJ/mol)	Coulombic Interaction (KJ/mol)	Total interactions (KJ/mol)
Voltage-Gated Sodium Channel – α2 (Nav1.2)			
Standard (Carbamazepine)	-99.8047	-11.793	-111.5977
Cyproheptadine	-117.832	-53.279	-171.111
Oxaprozin	-127.217	-8.5828	-135.7998
Pizotifen	-122.406	-21.6646	-144.0706
GABA receptor – α1 β1			
Standard (Clonazepam)	-35.405	-21.1496	-56.5546
Cyproheptadine	-41.7388	-14.1175	-55.8563
Oxaprozin	-47.8904	-16.6913	-64.5817
Pizotifen	-58.7435	-29.3292	-115.0727
Voltage-Gated Calcium Channel – α1G (Cav3.1)			
Standard (Pregabalin)	-62.8288	-2.68518	-65.51398
Cyproheptadine	-107.54	-81.9922	-189.5322
Oxaprozin	-118.768	-7.07558	-125.84350
Pizotifen	-117.041	-47.3968	-158.4378

For Nav1.2, the standard drug Carbamazepine had a total interaction energy of -111.5977 kJ/mol, while Cyproheptadine showed the most favourable energy at -171.111 kJ/mol, mainly due to stronger van der Waals (-117.832 kJ/mol) and Coulombic (-53.279 kJ/mol) interactions. Both Pizotifen (-144.0706 kJ/mol) and Oxaprozin (-135.7998 kJ/mol) also demonstrated better binding than the standard. In the GABA α1–β1 receptor system, Pizotifen again was the most stable with an interaction energy of -115.0727 kJ/mol, followed by Oxaprozin (-64.5817 kJ/mol). Cyproheptadine showed a slightly weaker interaction (-55.8563 kJ/mol) compared to the standard drug Clonazepam (-56.5546 kJ/mol), indicating receptor-specific affinity differences. Regarding Cav3.1, Pregabalin, the standard drug, had the weakest total interaction energy (-65.51398 kJ/mol), whereas Cyproheptadine had the most favourable at -189.5322 kJ/mol, mainly due to a strong Coulombic contribution (-81.9922 kJ/mol). Pizotifen (-158.4378 kJ/mol) and Oxaprozin (-125.8435 kJ/mol) also bound more strongly than Pregabalin. These results align with previous analyses showing Pizotifen and Oxaprozin exhibited more stable RMSD and radius of gyration values, suggesting tight, persistent binding during the simulation.

The comprehensive interaction energy profile designates Pizotifen as the most promising ligand across all receptors, owing to its sustained, high-affinity interactions, with Oxaprozin ranking subsequently. Although Cyproheptadine demonstrated lower affinity within the GABA complex, its elevated interaction energies with Nav1.2 and Cav3.1 render it a compelling candidate for drug repurposing. In conclusion, these interaction energies underscore the potential of all three pharmaceuticals to serve as efficacious modulators of vital neurological targets implicated in epilepsy.

### Hydrogen bond (H-bond)

**Fig. 11:**
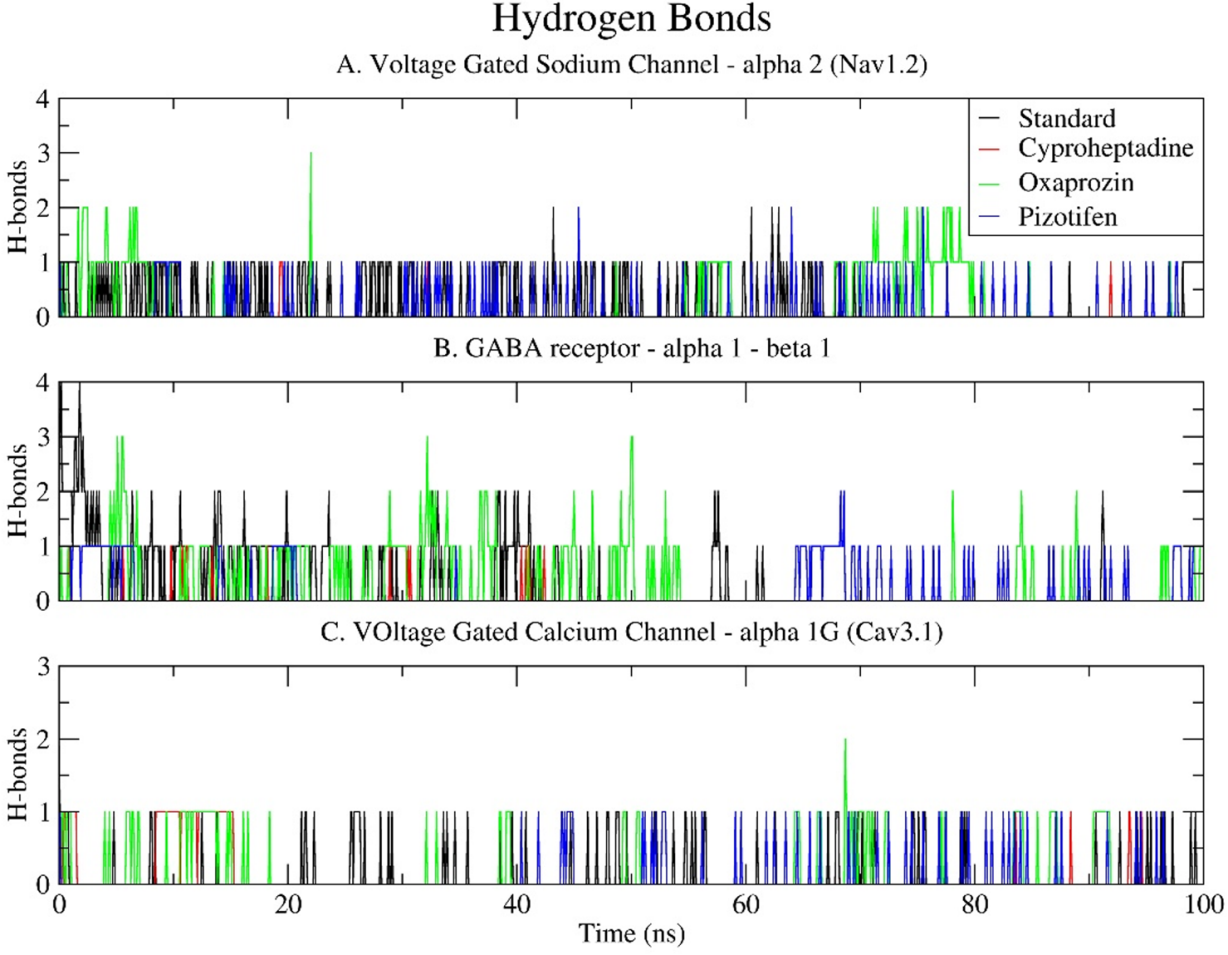
Time series plot depicting the number of hydrogen bonds (H-bonds) formed between the selected epilepsy proteins: (**A**) Voltage-gated sodium channel α2 (Nav1.2); (**B**) GABA receptor α1-β1; and (**C**) Voltage-gated calcium channel α1G (Cav3.1) and selected drugs (Standard, Cyproheptadine, Oxaprozin, Pizotifen) over a 100 ns simulation.

The analysis of hydrogen bonding provided critical insights into the stability and specificity of ligand–receptor interactions throughout the 100 ns molecular dynamics simulation. Among the compounds examined, Oxaprozin consistently demonstrated the highest number of hydrogen bonds across all three target receptors, indicating robust and persistent polar interactions within the binding sites. Specifically, with Nav1.2, Oxaprozin averaged 1.342 hydrogen bonds, reaching a peak of three bonds around 22 ns, although it predominantly maintained a single hydrogen bond during the simulation. Conversely, the standard drug Carbamazepine exhibited a significantly lower average of 0.199 hydrogen bonds, with sporadic formation of up to two hydrogen bonds during the mid-phase of the simulation. Both Cyproheptadine and Pizotifen formed negligible hydrogen bonds with Nav1.2 (0.007 and 0.027, respectively), which correlates with their lower interaction energies and suggests a primarily hydrophobic or non-polar binding mode (Fig. 11A).

The GABA receptor α1–β1 complex further corroborated the superior hydrogen bonding capacity of Oxaprozin, which averaged 1.532 hydrogen bonds, reaching a peak of three hydrogen bonds during the initial and middle stages of the simulation. The standard drug Clonazepam demonstrated a moderate average of 0.296 hydrogen bonds, initially forming up to four bonds that gradually diminished to one or two bonds towards the end. Pizotifen established a single hydrogen bond only after 60 nanoseconds, with an overall average of 0.155, while Cyproheptadine was observed to form sporadic and infrequent hydrogen bonds (0.024 on average), thereby indicating its lower polar affinity and reduced binding stability (Fig. 11B). Regarding Cav3.1, hydrogen bond formation was generally less frequent across all ligands. Nonetheless, Oxaprozin exhibited better performance, with an average of 0.821 hydrogen bonds, typically maintaining one bond throughout the simulation. The standard drug Pregabalin, in conjunction with Cyproheptadine, formed an average of 0.086 hydrogen bonds, with these interactions being transient. Pizotifen demonstrated the least hydrogen bond activity, averaging 0.021, consistent with its fewer polar contacts despite possessing a favourable interaction energy profile (Fig. 11C).

Overall, the hydrogen bond analysis highlights Oxaprozin’s robust polar interactions with all target proteins, corroborating the findings from interaction energy and RMSF analyses. This consistent hydrogen-bond engagement may contribute to maintaining stable binding and potentially modulate receptor conformation allosterically, thereby enhancing its potential as a compelling drug repurposing candidate for neurological targets.

### Molecular mechanics Poisson–Boltzmann surface area (MM-PBSA)

**Fig. 12:**
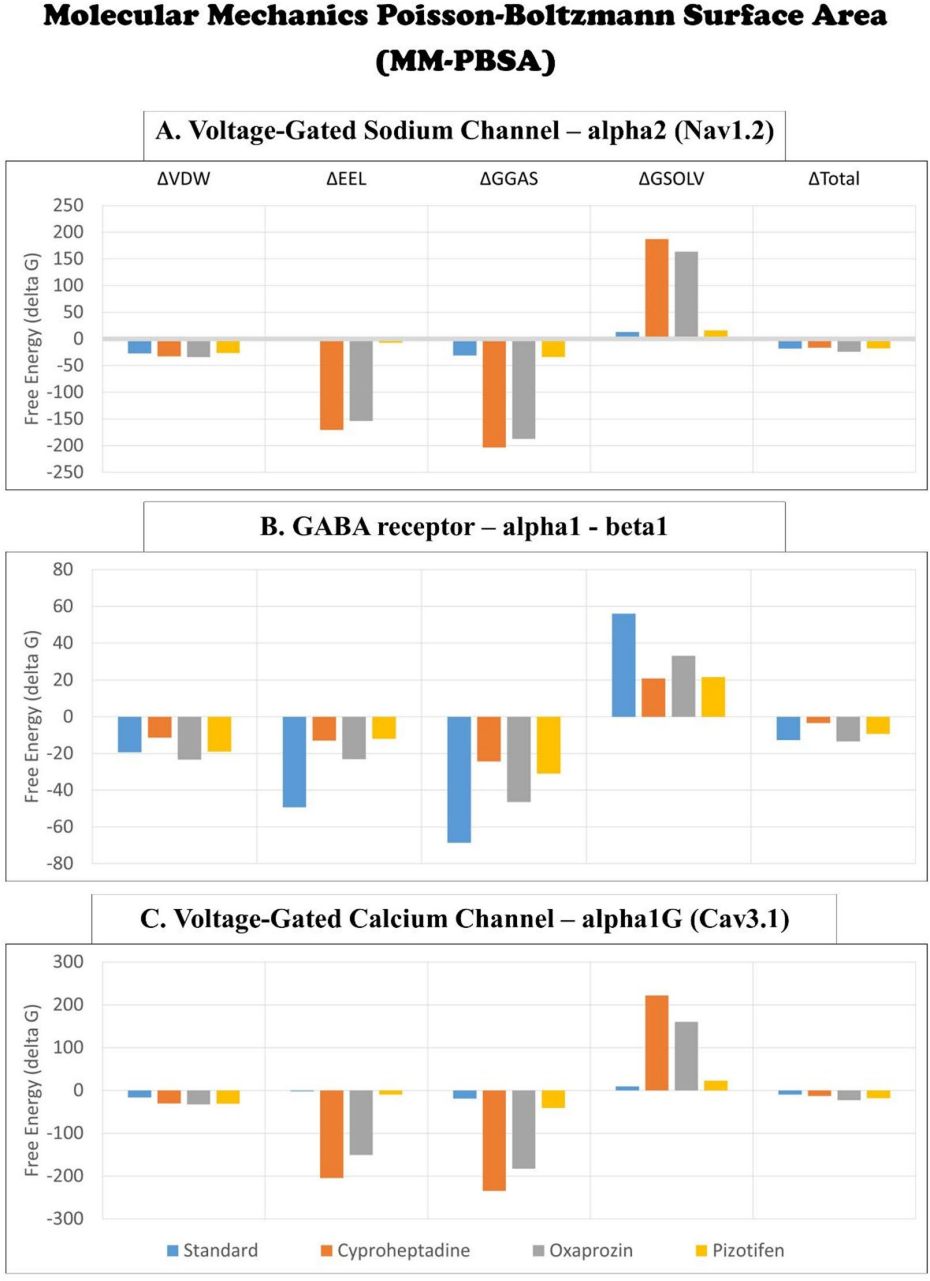
Time series plot depicting the Molecular Mechanics Poisson-Boltzmann Surface Area (MMPBSA) between the selected epilepsy proteins: (**A**) Voltage-gated sodium channel α2 (Nav1.2); (**B**) GABA receptor α1-β1; and (**C**) Voltage-gated calcium channel α1G (Cav3.1) and selected drugs (Standard, Cyproheptadine, Oxaprozin, Pizotifen) over a 100 ns simulation.

The MMPBSA method was employed to estimate the binding free energies of the selected drug–receptor complexes, offering insights into the thermodynamic stability and affinity of each ligand. The binding free energy components: van der Waals energy (ΔVDW), electrostatic energy (ΔEEL), gas-phase energy (ΔG_GAS = ΔBonds + ΔAngle + ΔDihedral + ΔVDW + ΔEEL), and solvation energy (ΔG_SOLV = ΔEGB + ΔESURF) were carefully evaluated. For the Nav1.2 receptor, Oxaprozin achieved the lowest ΔG_Total of − 23.98 kcal/mol, outperforming the standard drug Carbamazepine (-18.10 kcal/mol) and the investigational drugs Pizotifen (-17.78 kcal/mol) and Cyproheptadine (-16.35 kcal/mol). Notably, Oxaprozin showed the most favourable ΔVDW and ΔG_SOLV values, reflecting strong hydrophobic and solvent-based interactions (Fig. 12A; Table 6). In the GABA receptor α1–β1, Oxaprozin again demonstrated the most favourable ΔG_Total (-13.40 kcal/mol), followed by Clonazepam (-12.65 kcal/mol), Pizotifen (-9.32 kcal/mol), and Cyproheptadine (-3.41 kcal/mol). While Clonazepam exhibited the lowest ΔEEL and ΔG_GAS, Oxaprozin maintained the lowest ΔVDW and ΔG_SOLV, indicating its balanced contribution from both gas-phase and solvation components (Fig. 12B). For the Cav3.1, Oxaprozin recorded the lowest ΔG_Total of -22.94 kcal/mol, significantly outperforming Pizotifen (-17.93 kcal/mol) and Cyproheptadine (-13.13 kcal/mol). The standard drug Pregabalin showed the weakest interaction, with a ΔG_Total of only − 9.64 kcal/mol, highlighting Oxaprozin’s superior affinity for this calcium channel (Fig. 12C). Taken together, these findings strongly indicate that Oxaprozin demonstrates the most stable and energetically favourable binding profile across all three neuroreceptors, primarily driven by its robust van der Waals and solvation contributions. This reinforces its potential as a promising repurposed candidate for neurological targeting compared to the standard and other test drugs.

**Table 6:** The free binding energy (kcal/mol) between selected epilepsy receptor proteins and drugs using MMPBSA analysis.

Poisson Boltzmann	ΔVDW	ΔEEL	ΔG GAS	ΔG SOLV	ΔG Total
Voltage-Gated Sodium Channel – α2 (Nav1.2)					
Standard (Carbamazepine)	-27.38	-3.59	-30.97	12.87	-18.10
Cyproheptadine	-32.62	-170.84	-203.46	187.11	-16.35
Oxaprozin	-33.79	-153.70	-187.50	163.52	-23.98
Pizotifen	-26.53	-7.40	-33.93	16.15	-17.78
GABA receptor – α1 β1					
Standard (Clonazepam)	-19.40	-49.29	-68.68	56.04	-12.65
Cyproheptadine	-11.37	-12.95	-24.32	20.92	-3.41
Oxaprozin	-23.39	-23.09	-46.47	33.07	-13.40
Pizotifen	-18.98	-11.98	-30.97	21.65	-9.32
Voltage-Gated Calcium Channel – α1G (Cav3.1)					
Standard (Pregabalin)	-16.79	-2.15	-18.94	9.30	-9.64
Cyproheptadine	-30.40	-204.51	-234.91	221.78	-13.13
Oxaprozin	-32.44	-150.56	-183.01	160.07	-22.94
Pizotifen	-30.95	-9.99	-40.94	23.00	-17.93

Molecular dynamics simulations of the three target receptors —VGSC–α2 (Nav1.2), GABA receptor α1β1, and VGCC–α1G (Cav3.1) —indicated distinct dynamic behaviours and binding efficacies of the repurposed drugs Oxaprozin, Cyproheptadine, and Pizotifen. Initial structural assessments, such as RMSD, RMSF, Rg, and SASA, demonstrated that Oxaprozin induced greater fluctuations and flexibility, particularly within Nav1.2 and Cav3.1, signifying moderate destabilisation, in contrast to the more stable conformations associated with Cyproheptadine and Pizotifen. Subsequent analyses involving hydrogen bonding, interaction energy profiles, and MMPBSA calculations revealed that Oxaprozin established more stable and energetically favourable interactions with all three receptors, exhibiting the strongest non-bonded interaction energies and the lowest binding free energies. These findings suggest that, although Oxaprozin may provoke local conformational changes or increased flexibility, such effects enhance its overall binding affinity, potentially via deeper pocket penetration or allosteric engagement. Pizotifen, characterised by stable dynamics and favourable energetic profiles, ranked second overall, effectively balancing structural stability with consistent receptor binding. Cyproheptadine, while displaying dynamic stability, exhibited weaker hydrogen bonding and less favourable binding energies, particularly within the GABA complex, thereby ranking third. In conclusion, despite moderate fluctuations, Oxaprozin emerged as the most effective ligand, followed by Pizotifen and Cyproheptadine, underscoring their varied potentials as repurposed agents for modulating neuronal ion channels and receptors.

## Conclusion

A comprehensive in-silico drug repositioning approach was applied to screen the entire DrugBank database of molecules for identifying a new multi-targeted therapeutic option for epilepsy. A total of 2769 FDA-approved drugs were primarily screened for blood-brain permeability and structural similarity with currently marketed AEDs. The Voltage-Gated Sodium channel – α2, GABA receptor α1 β1, and Voltage-Gated Calcium channel – α1G, were selected as therapeutic target proteins for molecular docking studies using Carbamazepine, Clonazepam, and Pregabalin, as standard reference drugs, respectively. Only 46 marketed drugs, in common, showed higher binding affinities than the selected standard drugs. The binding pocket analyses and text mining studies narrowed our findings to three drug compounds, namely, Oxaprozin, Pizotifen, and Cyproheptadine, as potential candidates for drug repurposing as a multitarget treatment for epilepsy. The molecular dynamics simulation study revealed that all three selected drug compounds exhibit stable and strong binding to all three studied therapeutic target proteins. Oxaprozin was observed to show the strongest binding affinity and stability, followed by Pizotifen and Cyproheptadine. We believe that the in-silico simulation studies in our study provide robust hypothesis-generating mechanistic evidence for ligand–pocket engagement. Furthermore, structure refinement—encompassing pharmacophore modelling, scaffold modification, and fragment-based design—along with experimental validation, will be crucial in improving efficacy and reducing adverse effects, thereby supporting the clinical translation of multitarget therapies for epilepsy.

## Supplementary Information

Below is the link to the electronic supplementary material.

Supplementary Material 1

Supplementary Material 2

Supplementary Material 3

Supplementary Material 4

Supplementary Material 5

Supplementary Material 6

## Data Availability

All relevant data are provided in the supplementary files.

## Abbreviations

AEDs: Anti-epileptic Drugs
BBB: Blood Brain Barrier
GABA-R: Gamma-aminobutyric acid receptor
Rg: Radius of Gyration
RMSD: Root Mean Square Deviation
RMSF: Root Mean Square Fluctuations
SASA: Solvent Accessible Surface Area
VGCC: Voltage-gated calcium channel
VGSC: Voltage-gated sodium channel

## References

[1] Klein, P. et al. Repurposed molecules for antiepileptogenesis: missing an opportunity to prevent epilepsy? *Epilepsia* **61**, 359–386 (2020). 32196665 10.1111/epi.16450 PMC8317585

[2] Thijs, R. D., Surges, R., O’Brien, T. J. & Sander, J. W. Epilepsy in adults. *Lancet* **393**, 689–701 (2019). 30686584 10.1016/S0140-6736(18)32596-0

[3] Lossin, C., Wang, D. W., Rhodes, T. H., Vanoye, C. G. & George, A. L. Molecular basis of an inherited epilepsy. *Neuron* **34**, 877–884 (2002). 12086636 10.1016/s0896-6273(02)00714-6

[4] Annegers, J. F. & Rocca, W. A. Causes of epilepsy: Contributions of the rochester epidemiology project. in *Mayo Clin Proc* 71, 570–575 (Elsevier, 1996). 10.4065/71.6.570 8642886

[5] Galindo-Mendez, B., Mayor, L. C., Velandia-Hurtado, F. & Calderon-Ospina, C. Failure of antiepileptic drugs in controlling seizures in epilepsy: what do we do next? *Epilepsy Behav. Case Rep.* **4**, 6–8 (2015). 26101746 10.1016/j.ebcr.2015.03.004 PMC4454787

[6] Liu, Z. et al. In Silico drug repositioning-what we need to know. *Drug Discov Today*. **18**, 110–115 (2013). 22935104 10.1016/j.drudis.2012.08.005

[7] Ashburn, T. T. & Thor, K. B. Drug repositioning: identifying and developing new uses for existing drugs. *Nat. Rev. Drug Discov*. **3**, 673–683 (2004). 15286734 10.1038/nrd1468

[8] Jourdan, J. P., Bureau, R., Rochais, C. & Dallemagne, P. Drug repositioning: a brief overview. *J. Pharm. Pharmacol.* **72**, 1145–1151 (2020). 32301512 10.1111/jphp.13273 PMC7262062

[9] Tolete, P. et al. Lorcaserin therapy for severe epilepsy of childhood onset. *Neurology* **91**, 837–839 (2018). 30258026 10.1212/WNL.0000000000006432 PMC6207415

[10] Wishart, D. S. et al. DrugBank 5.0: A major update to the drugbank database for 2018. *Nucleic Acids Res.* **46**, D1074–D1082 (2018). 29126136 10.1093/nar/gkx1037 PMC5753335

[11] Kumar, P. et al. A machine Learning-based online tool for Blood-Brain barrier (BBB) permeability prediction. *CNS Neurol. Disord Drug Targets*. **24**, 1–9 (2024). 10.2174/0118715273328174241007060331 39415574

[12] Cheng, F. et al. AdmetSAR: A comprehensive source and free tool for assessment of chemical ADMET properties. *J. Chem. Inf. Model.* **52**, 3099–3105 (2012). 23092397 10.1021/ci300367a

[13] Shaker, B. et al. LightBBB: computational prediction model of blood-brain-barrier penetration based on LightGBM. *Bioinformatics* **37**, 1135–1139 (2021). 33112379 10.1093/bioinformatics/btaa918

[14] Hurle, M. R. et al. Computational drug repositioning: from data to therapeutics. *Clin. Pharmacol. Ther.* **93**, 335–341 (2013). 23443757 10.1038/clpt.2013.1

[15] Ekins, S., Williams, A. J., Krasowski, M. D. & Freundlich, J. S. In Silico repositioning of approved drugs for rare and neglected diseases. *Drug Discov Today*. **16**, 298–310 (2011). 21376136 10.1016/j.drudis.2011.02.016

[16] Kumar, P., Sheokand, D., Saini, V. & Kumar, A. In-silico identification and analysis of hub proteins for designing novel First-line Anti-seizure medications. *Lett. Drug Des. Discov*. **20**, 662–673 (2022).

[17] Backman, T. W. H., Cao, Y. & Girke, T. ChemMine tools: an online service for analyzing and clustering small molecules. *Nucleic Acids Res.* **39**, W486–W491 (2011). 21576229 10.1093/nar/gkr320 PMC3125754

[18] Cao, Y., Charisi, A., Cheng, L. C., Jiang, T. & Girke, T. ChemmineR: A compound mining framework for R. *Bioinformatics* **24**, 1733–1734 (2008). 18596077 10.1093/bioinformatics/btn307 PMC2638865

[19] Silvestro, S., Mammana, S., Cavalli, E., Bramanti, P. & Mazzon, E. Use of Cannabidiol in the treatment of epilepsy: efficacy and security in clinical trials. *Molecules* **24**, 1459 (2019). 31013866 10.3390/molecules24081459 PMC6514832

[20] Beydoun, A. et al. Current role of carbamazepine and oxcarbazepine in the management of epilepsy. *Seizure* **83**, 251–263 (2020). 33334546 10.1016/j.seizure.2020.10.018

[21] Dokkedal-Silva, V. et al. Indications, side Effects, and potential for nonmedical use. *Harv. Rev. Psychiatry*. **27**, 279–289 (2019). 31385811 10.1097/HRP.0000000000000227

[22] Hebeisen, S. et al. Eslicarbazepine and the enhancement of slow inactivation of voltage-gated sodium channels: A comparison with carbamazepine, oxcarbazepine and lacosamide. *Neuropharmacology* **89**, 122–135 (2015). 25242737 10.1016/j.neuropharm.2014.09.008

[23] Brigo, F. & Igwe, S. C. Ethosuximide, sodium valproate or lamotrigine for absence seizures in children and adolescents. *Cochrane Database of Systematic Reviews* (2017). 10.1002/14651858.CD003032.pub3 PMC6464603 28195639

[24] Markota, M. & Morgan, R. J. Treatment of generalized anxiety disorder with Gabapentin. *Case Rep. Psychiatry*. **2017**, 1–4 (2017). 10.1155/2017/6045017 PMC5745655 29387502

[25] Rogawski, M. A., Tofighy, A., White, H. S., Matagne, A. & Wolff, C. Current Understanding of the mechanism of action of the antiepileptic drug lacosamide. *Epilepsy Res.* **110**, 189–205 (2015). 25616473 10.1016/j.eplepsyres.2014.11.021 PMC13325623

[26] Stockburger, C. et al. A mitochondrial role of SV2a protein in aging and alzheimer’s disease: studies with Levetiracetam. *J. Alzheimer’s Disease*. **50**, 201–215 (2016). 26639968 10.3233/JAD-150687

[27] Krauss, G. L. et al. Levetiracetam treatment of idiopathic generalised epilepsy. *Seizure* **12**, 617–620 (2003). 14630506 10.1016/s1059-1311(03)00139-0

[28] Mruk, A. L., Garlitz, K. L. & Leung, N. R. Levetiracetam in neonatal seizures: A review. *J. Pediatr. Pharmacol. Ther.* **20**, 76–89 (2015). 25964725 10.5863/1551-6776-20.2.76 PMC4418685

[29] Hansen, C. C., Ljung, H., Brodtkorb, E. & Reimers, A. Mechanisms underlying aggressive behavior induced by antiepileptic drugs: Focus on topiramate, levetiracetam, and perampanel. *Behav. Neurol.* (2018). 10.1155/2018/2064027 PMC6276511 30581496

[30] Alles, S. R. A., Cain, S. M. & Snutch, T. P. Pregabalin as a pain therapeutic: beyond calcium channels. *Front. Cell. Neurosci.* **14**, 83 (2020). 32351366 10.3389/fncel.2020.00083 PMC7174704

[31] Manjushree, N., Chakraborty, A., Shashidhar, K. & Narayanaswamy, S. A review of the drug Pregabalin. *Int. J. Basic. Clin. Pharmacol.* 601–605. 10.18203/2319-2003.ijbcp20150359 (2015).

[32] Perucca, E., Cloyd, J., Critchley, D. & Fuseau, E. Rufinamide: Clinical pharmacokinetics and concentration-response relationships in patients with epilepsy. *Epilepsia* **49**, 1123–1141 (2008). 18503564 10.1111/j.1528-1167.2008.01665.x

[33] Piplani, S., Verma, P. K. & Kumar, A. Neuroinformatics analyses reveal GABAt and SSADH as major proteins involved in anticonvulsant activity of valproic acid. *Biomed. Pharmacotherapy*. **81**, 402–410 (2016). 10.1016/j.biopha.2016.04.036 27261619

[34] Romoli, M. et al. Valproic acid and epilepsy: from molecular mechanisms to clinical evidences. *Curr. Neuropharmacol.* **17**, 926–946 (2018). 10.2174/1570159X17666181227165722 PMC7052829 30592252

[35] Zanatta, G. et al. Valproic acid interactions with the NavMs voltage-gated sodium channel. *Proc. Natl. Acad. Sci. U S A*. **116**, 26549–26554 (2019). 31822620 10.1073/pnas.1909696116 PMC6936711

[36] Luszczki, J. J., Ratnaraj, N., Patsalos, P. N. & Czuczwar, S. J. Isobolographic and behavioral characterizations of interactions between Vigabatrin and Gabapentin in two experimental models of epilepsy. *Eur. J. Pharmacol.* **595**, 13–21 (2008). 18708046 10.1016/j.ejphar.2008.07.051

[37] Biton, V. Clinical Pharmacology and mechanism of action of Zonisamide. *Clin. Neuropharmacol.* **30**, 230–240 (2007). 17762320 10.1097/wnf.0b013e3180413d7d

[38] Pan, X. et al. Molecular basis for pore Blockade of human Na + channel Na v 1.2 by the m-conotoxin KIIIA. *Sci. (1979)*. **363**, 1309–1313 (2019). 10.1126/science.aaw2999 30765605

[39] Masiulis, S. et al. GABAA receptor signalling mechanisms revealed by structural Pharmacology. *Nature* **565**, 454–459 (2019). 30602790 10.1038/s41586-018-0832-5 PMC6370056

[40] Zhao, Y. et al. Cryo-EM structures of Apo and antagonist-bound human Cav3.1. *Nature* **576**, 492–497 (2019). 31766050 10.1038/s41586-019-1801-3

[41] Arnold, K., Bordoli, L., Kopp, J. & Schwede, T. The SWISS-MODEL workspace: A web-based environment for protein structure homology modelling. *Bioinformatics* **22**, 195–201 (2006). 16301204 10.1093/bioinformatics/bti770

[42] Benkert, P., Tosatto, S. C. E. & Schomburg, D. QMEAN: A comprehensive scoring function for model quality assessment. *Proteins: Struct. Function Genet.* **71**, 261–277 (2008). 10.1002/prot.21715 17932912

[43] Agnihotry, S., Pathak, R. K., Singh, D. B., Tiwari, A. & Hussain, I. Protein structure prediction. *Bioinformatics: Methods Appl.* 177–188. 10.1016/B978-0-323-89775-4.00023-7 (2021).

[44] Eisenberg, D., Lüthy, R. & Bowie, J. U. VERIFY3D: assessment of protein models with three-dimensional profiles. *Methods Enzymol.* **277**, 396–404 (1997). 9379925 10.1016/s0076-6879(97)77022-8

[45] Wiederstein, M. & Sippl, M. J. ProSA-web: interactive web service for the recognition of errors in three-dimensional structures of proteins. *Nucleic Acids Res.* **35**, W407–W410 (2007). 17517781 10.1093/nar/gkm290 PMC1933241

[46] Macindoe, G., Mavridis, L., Venkatraman, V., Devignes, M. D. & Ritchie, D. W. HexServer: an FFT-based protein Docking server powered by graphics processors. *Nucleic Acids Res* **38** (2010). 10.1093/nar/gkq311 PMC2896144 20444869

[47] Delano, L. W. Pymol: an open-source molecular graphics tool. *CCP4 Newsl. Protein Crystallogr.* **40**, 82–92 (2002).

[48] Pettersen, E. F. et al. UCSF Chimera - A visualization system for exploratory research and analysis. *J. Comput. Chem.* **25**, 1605–1612 (2004). 15264254 10.1002/jcc.20084

[49] Morris, G. M. et al. Software news and updates AutoDock4 and AutoDockTools4: automated Docking with selective receptor flexibility. *J. Comput. Chem.* **30**, 2785–2791 (2009). 19399780 10.1002/jcc.21256 PMC2760638

[50] Kumar, P., Sheokand, D., Grewal, A., Saini, V. & Kumar, A. Clinical side-effects based drug repositioning for anti-epileptic activity. *J. Biomol. Struct. Dyn.* **42**, 1443–1454 (2024). 37042987 10.1080/07391102.2023.2199874

[51] Laskowski, R. A., Swindells, M. B. & LigPlot+ Multiple ligand-protein interaction diagrams for drug discovery. *J. Chem. Inf. Model.* **51**, 2778–2786 (2011). 21919503 10.1021/ci200227u

[52] Abraham, M. J. et al. High performance molecular simulations through multi-level parallelism from laptops to supercomputers. *SoftwareX* **1–2**, 19–25 (2015). Gromacs.

[53] Huang, J. et al. CHARMM36m: an improved force field for folded and intrinsically disordered proteins. *Nat. Methods*. **14**, 71–73 (2016). 27819658 10.1038/nmeth.4067 PMC5199616

[54] Valdés-Tresanco, M. S., Valdés-Tresanco, M. E., Valiente, P. A. & Moreno, E. Gmx_MMPBSA: A new tool to perform End-State free energy calculations with GROMACS. *J. Chem. Theory Comput.* **17**, 6281–6291 (2021). 34586825 10.1021/acs.jctc.1c00645

[55] Ghit, A., Assal, D., Al-Shami, A. S. & Hussein, D. E. E. GABAA receptors: structure, function, pharmacology, and related disorders. *J. Genet. Eng. Biotechnol.* **19**, 1–15 (2021). 10.1186/s43141-021-00224-0 PMC8380214 34417930

[56] Sigel, E. & Steinmann, M. E. Structure, function, and modulation of GABAA receptors. *J. Biol. Chem.* **287**, 40224–40231 (2012). 23038269 10.1074/jbc.R112.386664 PMC3504738

[57] Abdelsayed, M. & Sokolov, S. Voltage-gated sodium channels: pharmaceutical targets via anticonvulsants to treat epileptic syndromes. *Channels* **7**, 146–152 (2013). 23531742 10.4161/chan.24380 PMC3710341

[58] Docherty, R. J. & Farmer, C. E. The Pharmacology of voltage-gated sodium channels in sensory neurones. *Handb. Exp. Pharmacol.* **194**, 519–561 (2009). 10.1007/978-3-540-79090-7_15 19655117

[59] Lipkind, G. M. & Fozzard, H. A. Molecular model of anticonvulsant drug binding to the voltage-gated sodium channel inner pore. *Mol. Pharmacol.* **78**, 631–638 (2010). 20643904 10.1124/mol.110.064683 PMC2981395

[60] Ghit, A., Assal, D., Al-Shami, A. S. & Hussein, D. E. E. GABAA receptors: structure, function, pharmacology, and related disorders. *J. Genet. Eng. Biotechnol.* **19**, 123 (2021). 10.1186/s43141-021-00224-0 PMC8380214 34417930

[61] Goldschen-Ohm, M. P., Wagner, D. A. & Jones, M. V. Three arginines in the GABAA receptor binding pocket have distinct roles in the formation and stability of Agonist- versus Antagonist-Bound complexes. *Mol. Pharmacol.* **80**, 647 (2011). 21764985 10.1124/mol.111.072033 PMC3187534

[62] Padgett, C. L., Hanek, A. P., Lester, H. A., Dougherty, D. A. & Lummis, S. C. R. Unnatural amino acid mutagenesis of the GABAA receptor binding site residues reveals a novel Cation–π interaction between GABA and β2Tyr97. *J. Neurosci.* **27**, 886 (2007). 17251430 10.1523/JNEUROSCI.4791-06.2007 PMC2649369

[63] Phulera, S. et al. Cryo-EM structure of the benzodiazepine-sensitive α1β1γ2S tri-heteromeric GABAA receptor in complex with GABA. *Elife* 7 (2018). 10.7554/eLife.39383 PMC6086659 30044221

[64] Cunningham, K. L. & Littleton, J. T. Mechanisms controlling the trafficking, localization, and abundance of presynaptic Ca2 + channels. *Front. Mol. Neurosci.* **15**, 767 (2023). 10.3389/fnmol.2022.1116729 PMC9880069 36710932

[65] Katelaris, C. Comparative effects of Loratadine and Azatadine in the treatment of seasonal allergic rhinitis. *Asian Pac. J. Allergy Immunol.* **8**, 103–107 (1990). 1982614

[66] Welch, M. J., Meltzer, E. O., Estelle, F. & Simons, R. H1-antihistamines and the central nervous system. Histamine and H1-Antihistamines in allergic disease. *Second Ed. Revis. Expanded* 337–388. 10.3109/9780203910375-16 (2002). 12113223

[67] Hill, T. et al. Antidepressant use and risk of epilepsy and seizures in people aged 20 to 64 years: cohort study using a primary care database. *BMC Psychiatry* **15** (2015). 10.1186/s12888-015-0701-9 PMC4683813 26678837

[68] Bertrand, V. et al. Safety of Cyproheptadine, an orexigenic Drug. Analysis of the French National pharmacovigilance Data-Base and systematic review. *Front. Pediatr.* **9**, 712413 (2021). 34676184 10.3389/fped.2021.712413 PMC8525494

[69] Kalpaklioglu, F. & Baccioglu, A. Efficacy and safety of H1-Antihistamines: an update. *Antiinflamm Antiallergy Agents Med. Chem.* **11**, 230–237 (2013). 10.2174/1871523011202030230 23173575

[70] Winkler, J. L., Skovira, J. W. & Kan, R. K. Anticonvulsant efficacy of antihistamine Cyproheptadine in rats exposed to the chemical warfare nerve agent Soman. *Neurotoxicology* **58**, 153–160 (2017). 27988303 10.1016/j.neuro.2016.12.004

[71] Baumann, P. A. & Maitre, L. Neurobiochemical aspects of maprotiline (Ludiomil ^®^) action. *J. Int. Med. Res.* **7**, 391–400 (1979). 387493 10.1177/030006057900700511

[72] Barbaccia, M. L., Ravizza, L., Costa, E. & Maprotiline An antidepressant with an unusual Pharmacological profile. *J. Pharmacol. Exp. Ther.* **236**, 307–312 (1986). 3003338

[73] Johannessen Landmark, C., Henning, O. & Johannessen, S. I. Proconvulsant effects of antidepressants — What is the current evidence? *Epilepsy Behav.* **61**, 287–291 (2016). 26926001 10.1016/j.yebeh.2016.01.029

[74] Hoffman, B. F. & Wachsmuth, R. Maprotiline and seizures. *J. Clin. Psychiatry*. **43**, 117–118 (1982). 6801027

[75] Derry, S., Wiffen, P. J., Aldington, D. & Moore, R. A. Nortriptyline for neuropathic pain in adults. *Cochrane Database of Systematic Reviews* (2017). 10.1002/14651858.CD011209.pub2 PMC6485407 25569864

[76] Reinert, J. P., Veronin, M. A. & Medina, C. Tricyclic antidepressants in nociceptive and neuropathic pain: A review of their analgesic properties in combination with opioids. *J. Pharm. Technol.* **39**, 35–40 (2023). 36755751 10.1177/87551225221139699 PMC9899962

[77] Wiffen, P. J., Moore, R. A., Aldington, D. & Derry, S. Nortriptyline for neuropathic pain in adults. *Cochrane Database of Systematic Reviews* (2014). 10.1002/14651858.CD011209.pub2 PMC6485407 25569864

[78] Luchins, D. J., Oliver, A. P. & Wyatt, R. J. Seizures with antidepressants: an in vitro technique to assess relative risk. *Epilepsia* **25**, 25–32 (1984). 6692788 10.1111/j.1528-1157.1984.tb04151.x

[79] Lipper, B., Bell, A. & Gaynor, B. Recurrent hypotension immediately after seizures in Nortriptyline overdose. *Am. J. Emerg. Med.* **12**, 452–453 (1994). 8031432 10.1016/0735-6757(94)90060-4

[80] Peesa, J. P. et al. Oxaprozin prodrug as safer nonsteroidal anti-inflammatory drug: synthesis and Pharmacological evaluation. *Arch. Pharm. (Weinheim)*. **351**, 1700256 (2018). 10.1002/ardp.201700256 29283449

[81] Khatami, P., Mirazi, N., Khosravi, M. & Bananej, M. Nonsteroidal Anti-inflammatory drug Oxaprozin is beneficial against Seizure-induced memory impairment in an experimental model of seizures in rats: impact on oxidative stress and Nrf2/HO-1 signaling pathway. *J. Mol. Neurosci.* **72**, 880–887 (2022). 35084669 10.1007/s12031-022-01967-2

[82] Robbins, M. S. & Burch, R. Preventive migraine treatment. *CONTINUUM Lifelong Learn. Neurol.* **27**, 613–632 (2021). 10.1212/CON.0000000000000957 34048395

[83] Tronvik, E., Giri, S. & Young, W. Preventive treatment of migraine: Non-specific oral agents. *Handb. Clin. Neurol.* **199**, 67–86 (2024). 38307673 10.1016/B978-0-12-823357-3.00009-4

[84] Batista, F. L. A. et al. Anticonvulsant and anxiolytic-like potential of the essential oil from the ocimum Basilicum Linn leaves and its major constituent Estragole on adult zebrafish (Danio rerio). *Neurochem Int.* **178**, 105796 (2024). 38936553 10.1016/j.neuint.2024.105796

