# Supplementary material for: Computational drug repositioning approach to predict multi-target therapeutics for epilepsy
Source: Sci Rep. 2025 Dec 16;15:43927. doi: 10.1038/s41598-025-27625-2 (PMC12708636; doi:10.1038/s41598-025-27625-2)
Supplement: Supplementary file 6 — Supplementary Material 6 [file 41598_2025_27625_MOESM6_ESM.pdf]

Binding interaction

DrugBank ID<br>PubChem ID<br>Name	Voltage gated sodium channel α2<br>(Nav 1.2)	GABA Receptor α1-β1	Voltage gated calcium channel α1G<br>(Cav 3.1)
Standards	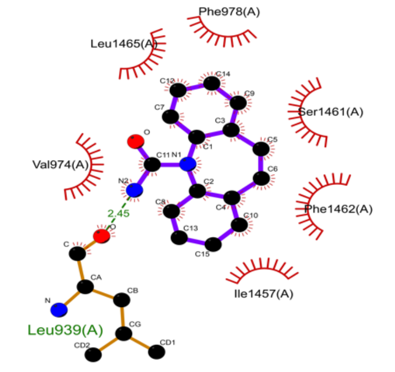	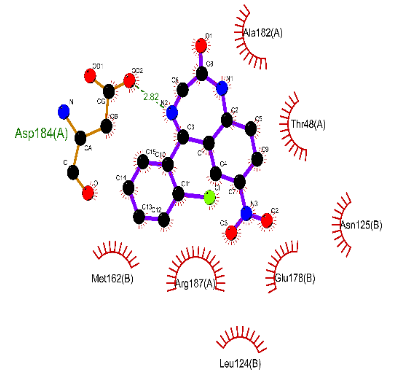	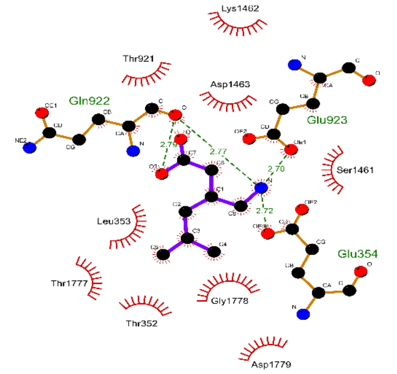
DB00192<br>52195<br>Indecainide	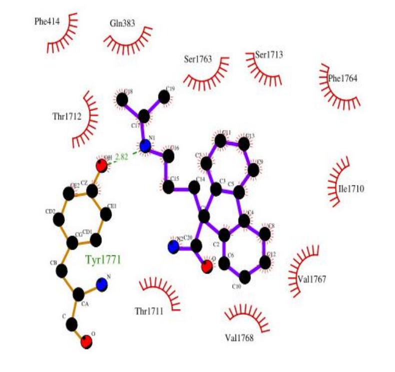	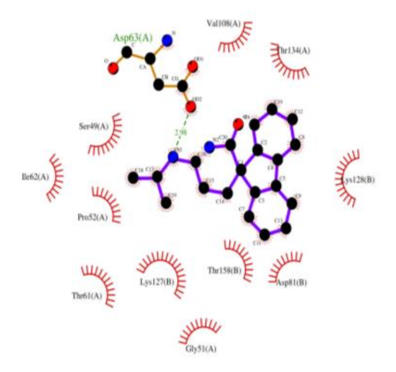	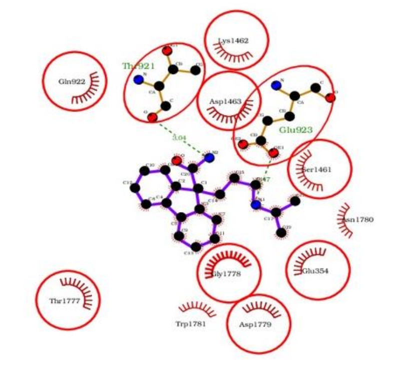
DB00321<br>2160<br>Amitriptyline	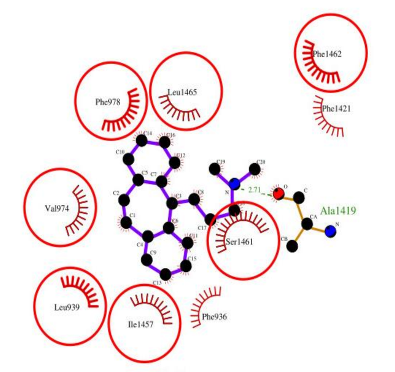	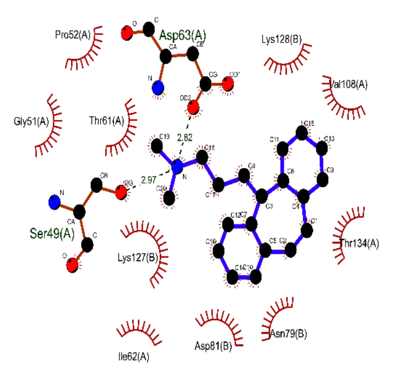	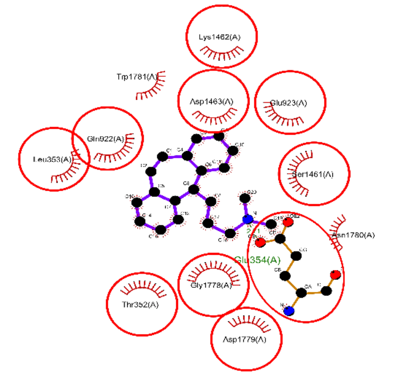
DB00340<br>4167<br>Metixene	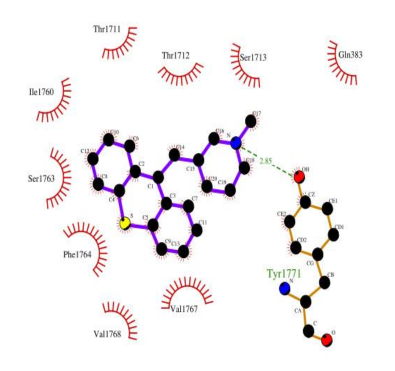	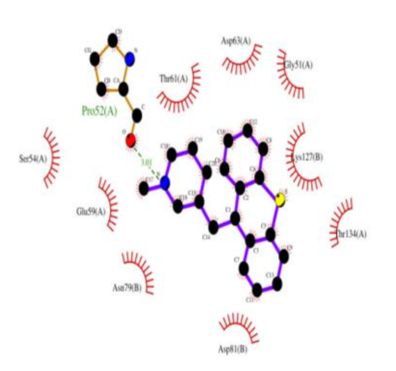	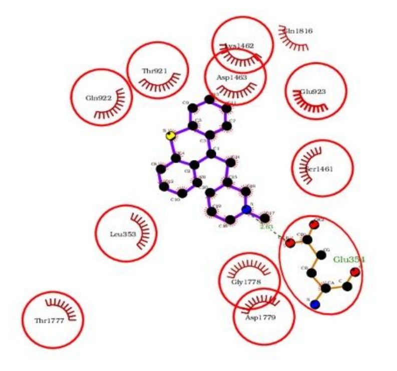

DB00344<br>4976<br>Protriptyline	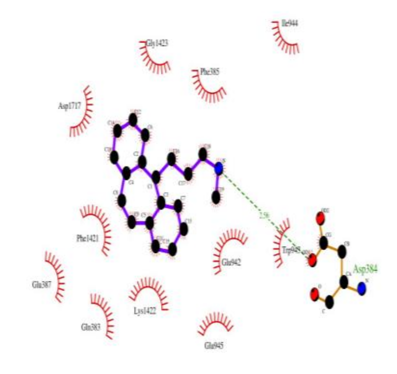	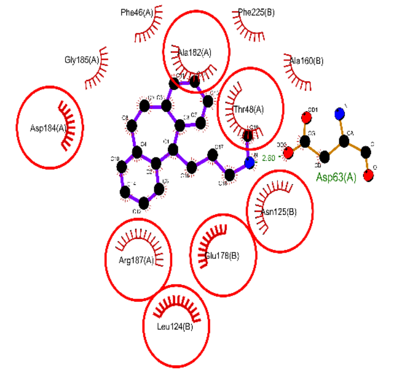	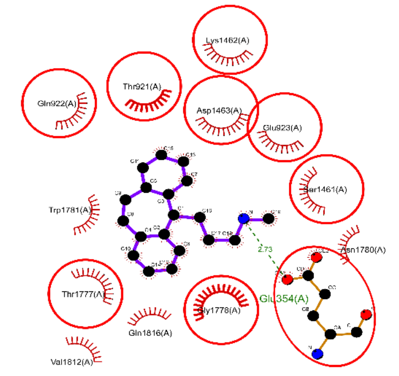
DB00427<br>5282443<br>Triprolidine	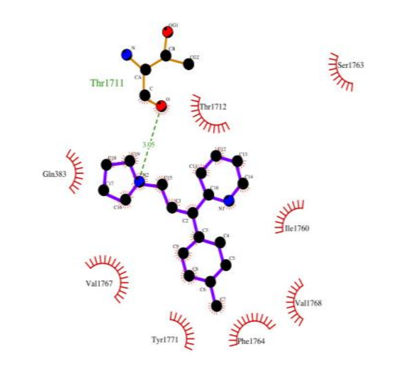	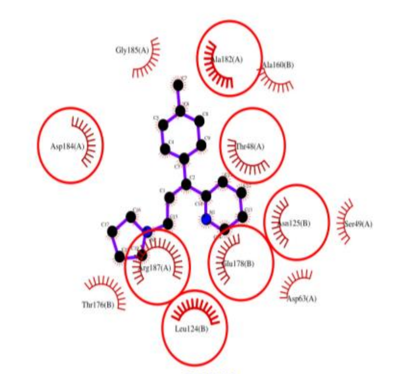	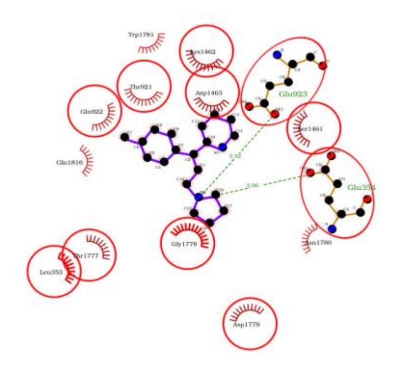
DB00434<br>2913<br>Cyproheptadine	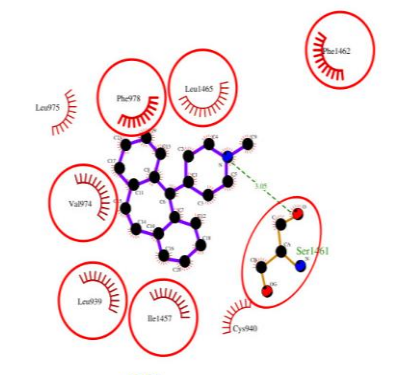	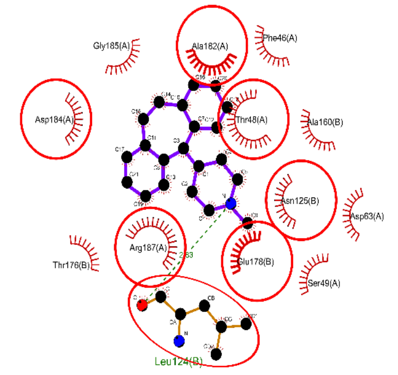	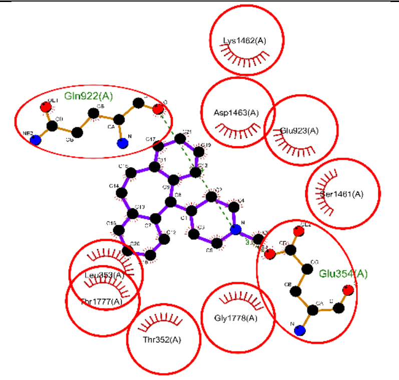
DB00486<br>5284592<br>Nabilone	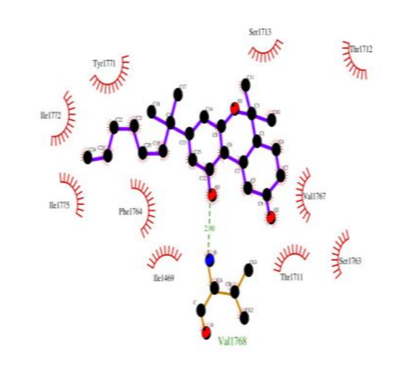	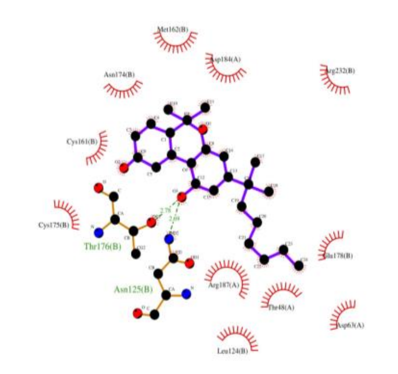	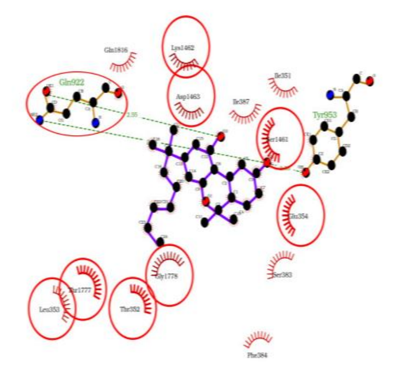
DB00496<br>444031<br>Darifenacin	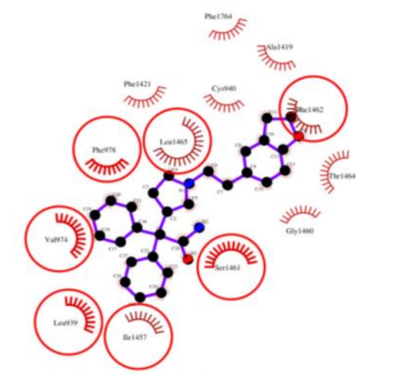	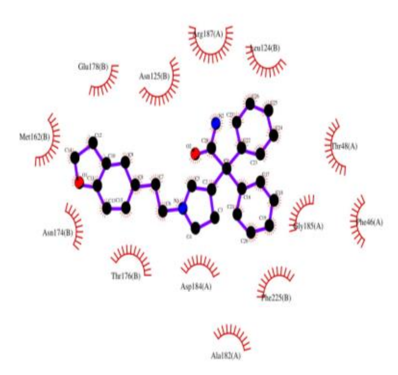	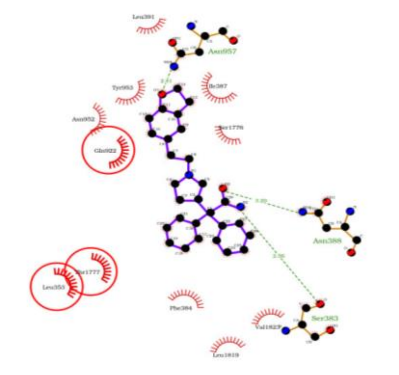

DB00540 4543 Nortriptyline	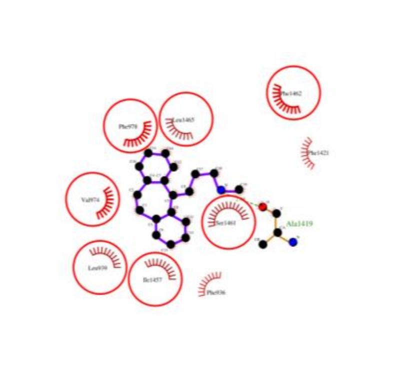	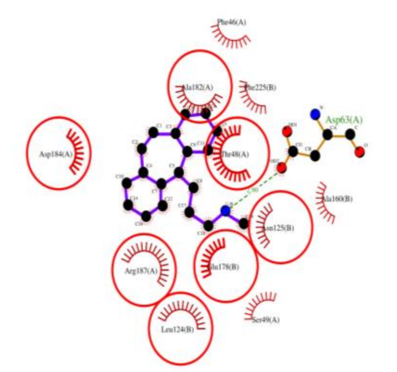	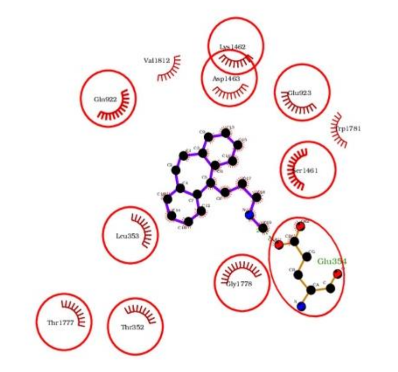
DB00561 3156 Doxapram	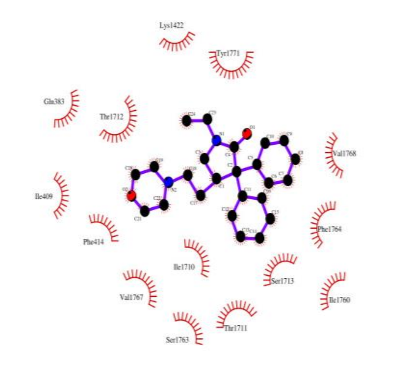	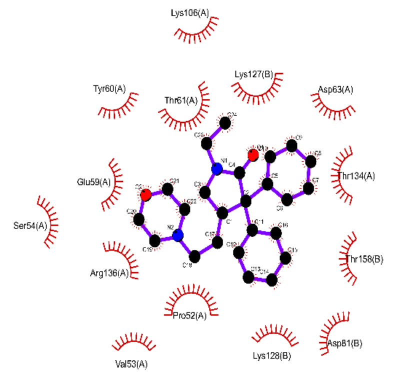	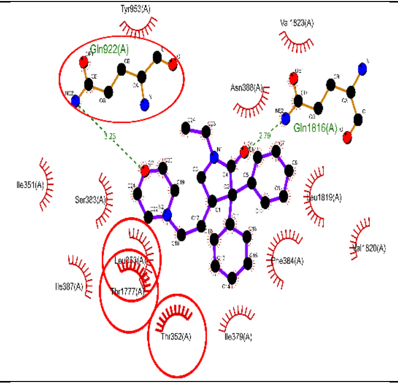
DB00568 2761 Cinnarizine	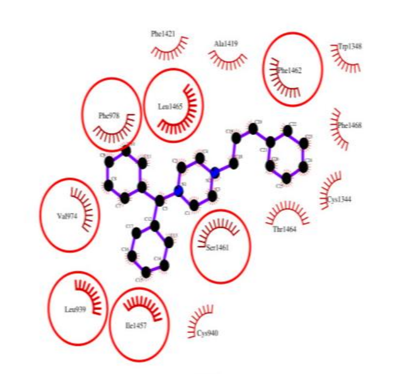	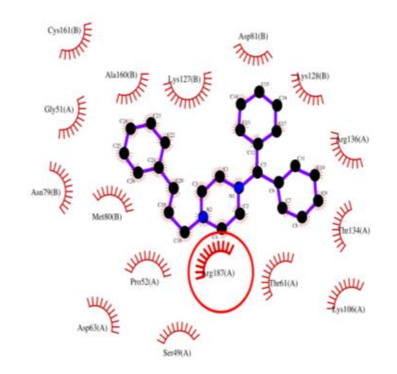	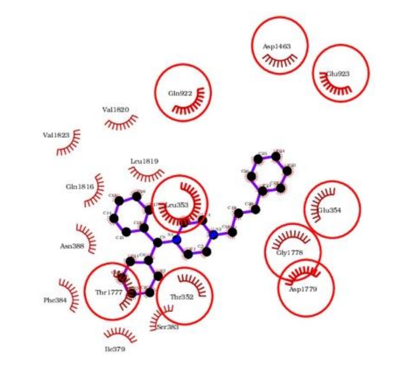
DB00693 16850 Fluorescein	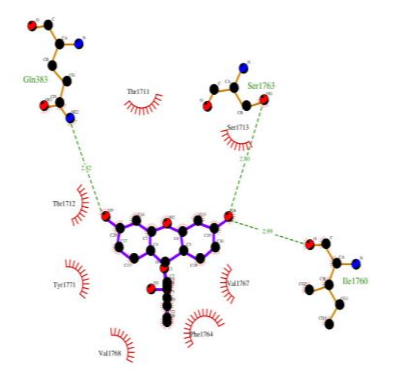	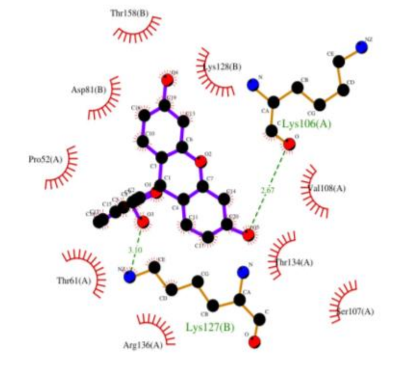	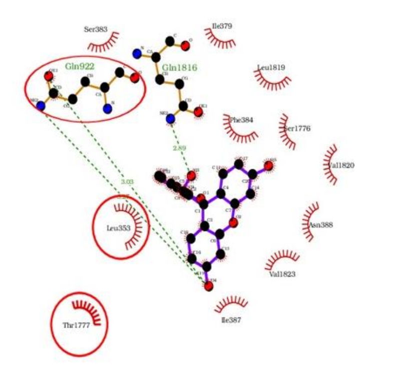
DB00719 19861 Azatadine	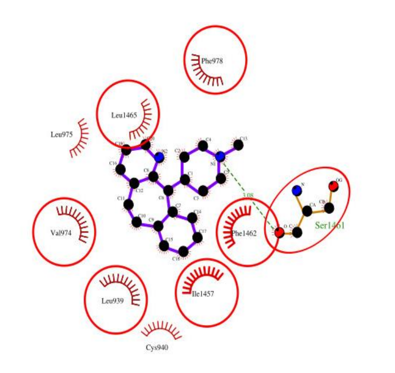	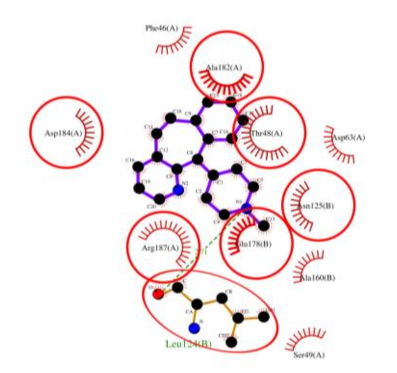	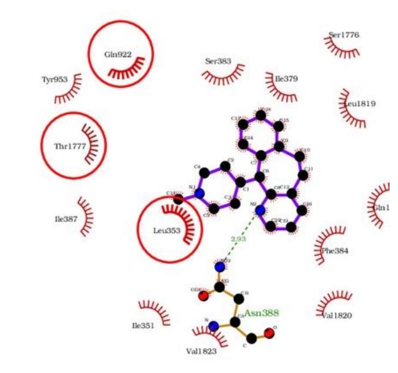

DB00850 4748 Perphenazine	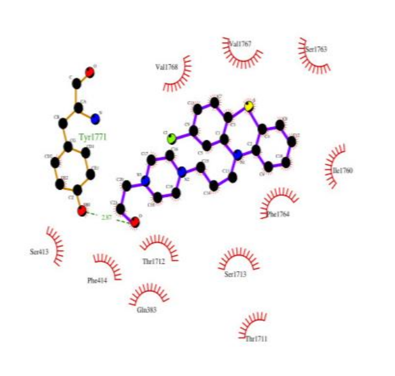	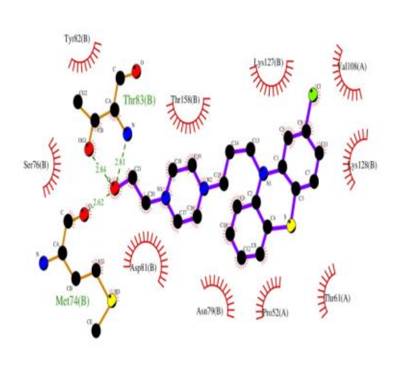	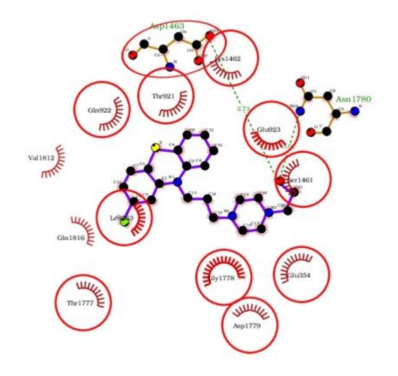
DB00920 3827 Ketotifen	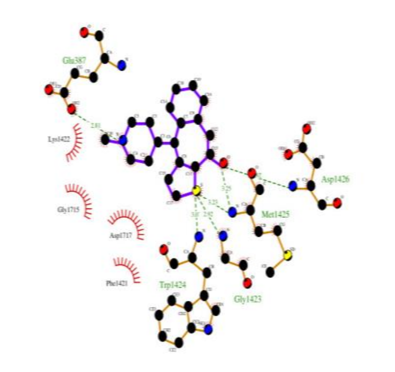	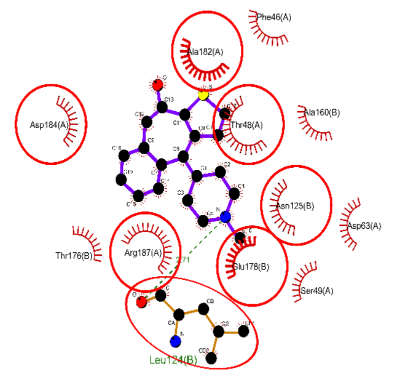	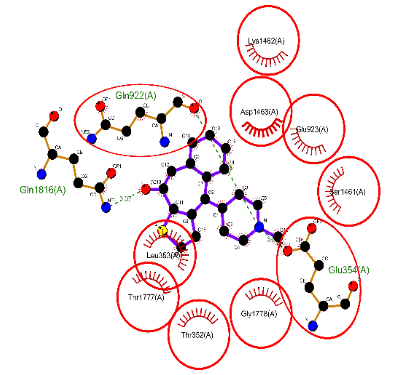
DB00924 2895 Cyclobenzaprine	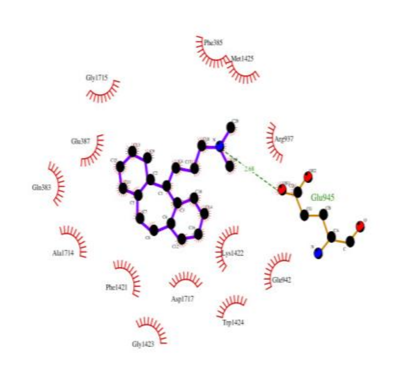	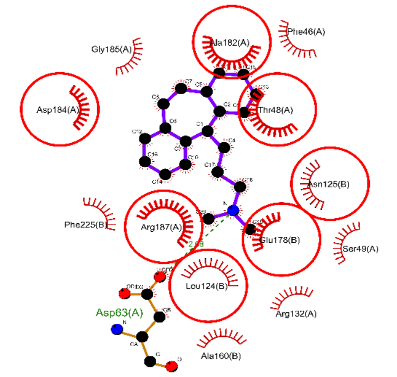	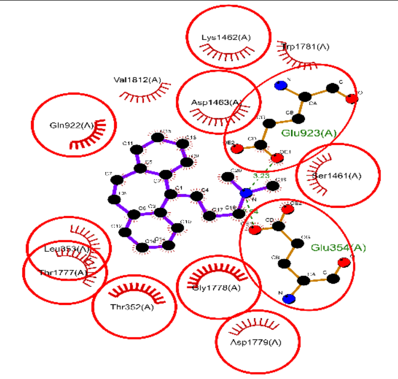
DB00933 4078 Mesoridazine	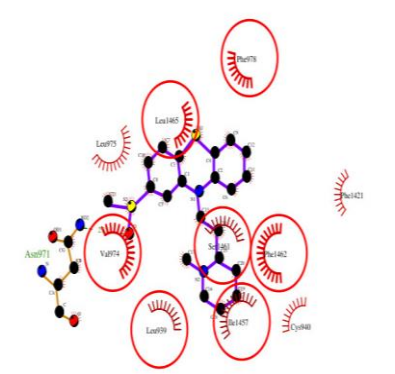	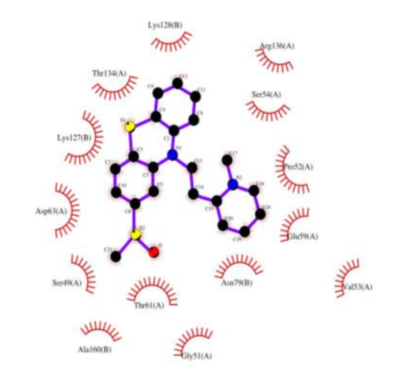	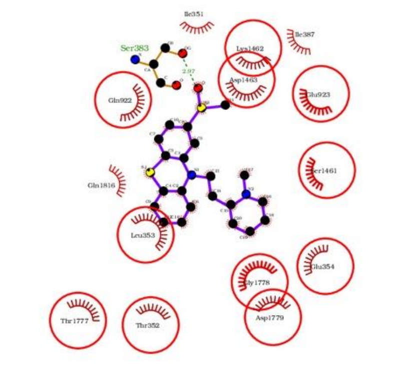
DB00934 4011 Maprotiline	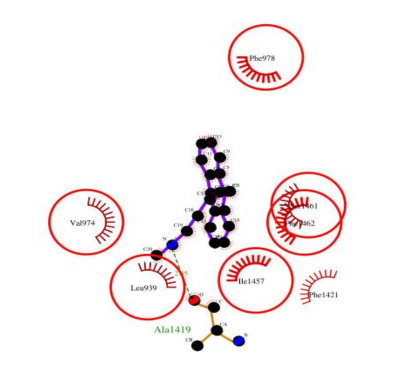	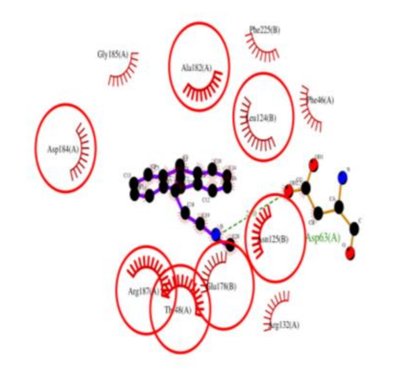	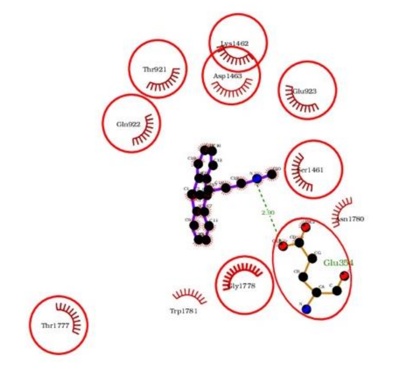

DB00972 2267 Azelastine

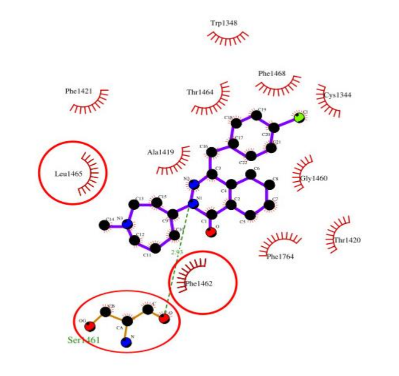

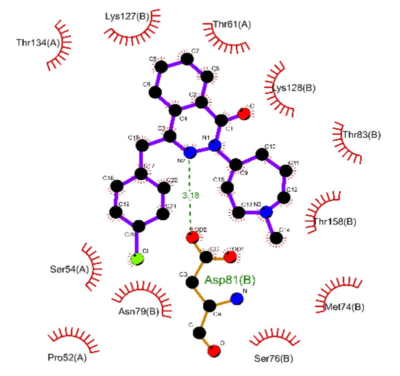

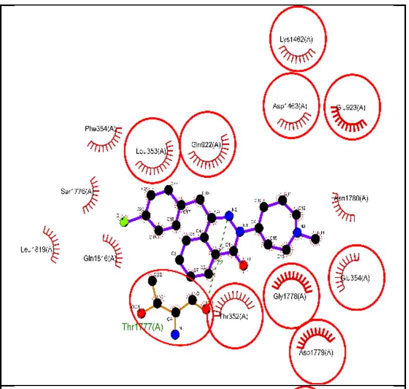

DB00991 4614 Oxaprozin

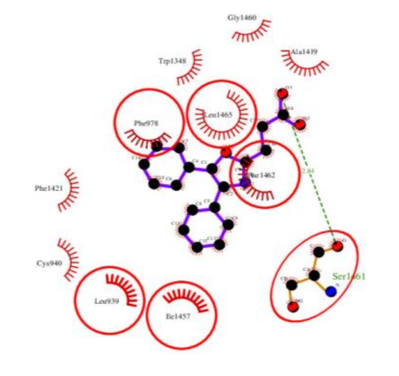

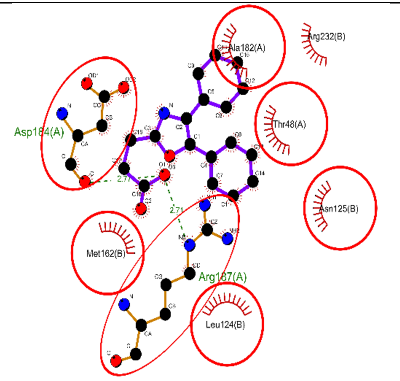

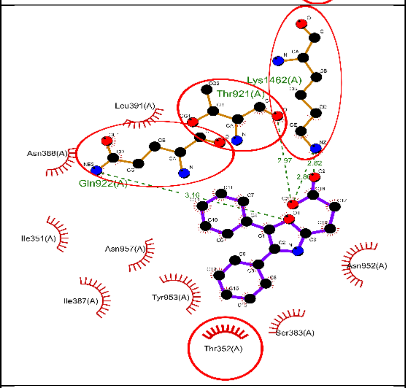

DB01009 3825 Ketoprofen

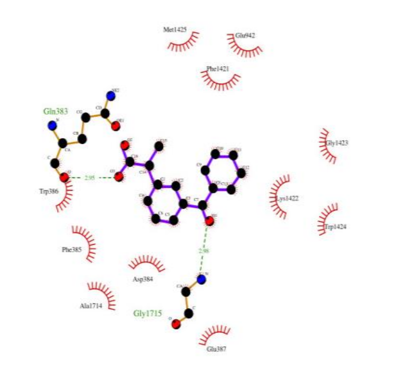

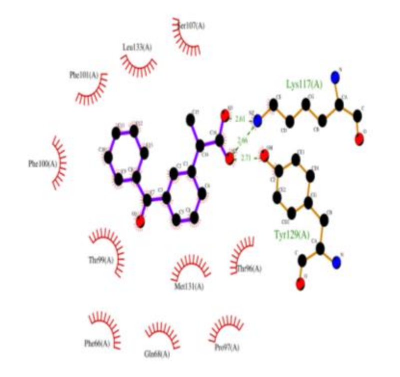

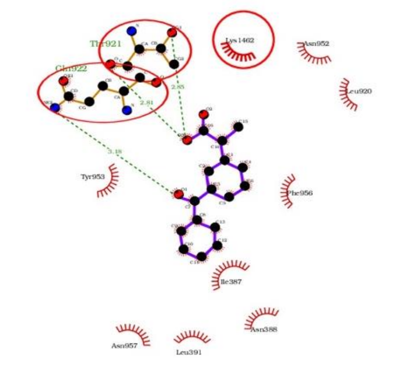

DB01100 16362 Pimozide

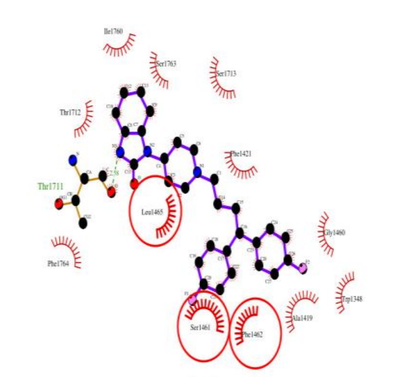

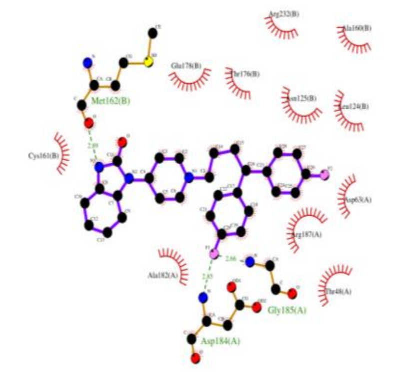

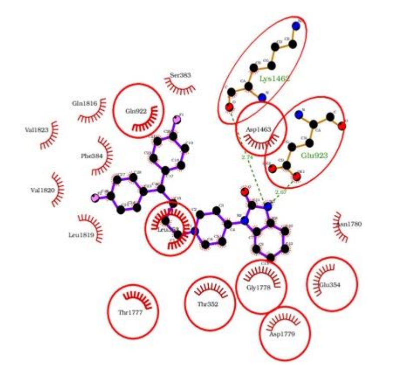

DB01142 667468 Doxepin

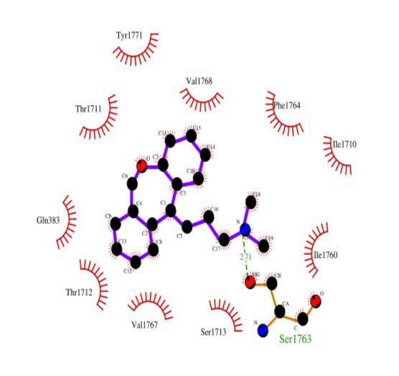

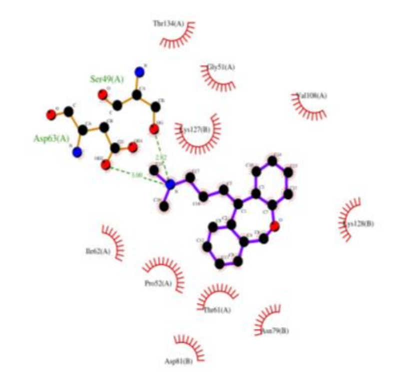

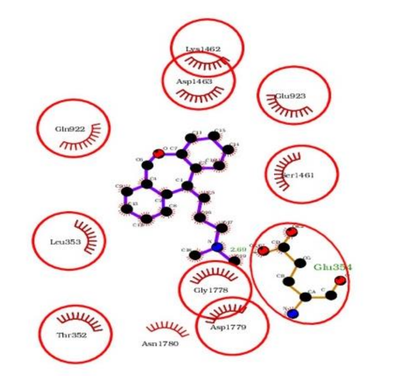

DB01146 3103 Diphenylpyraline	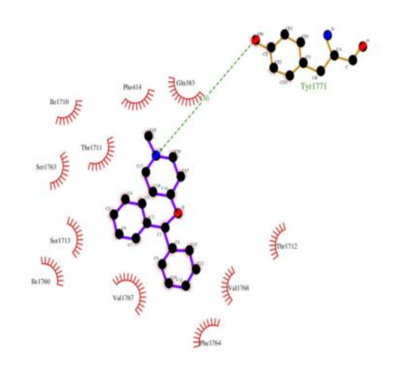	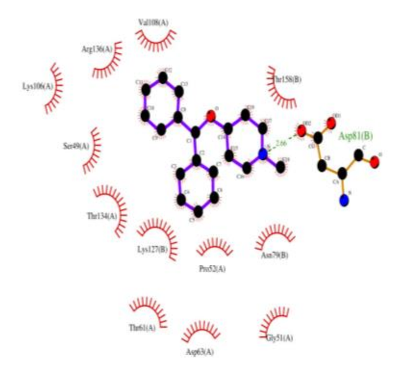	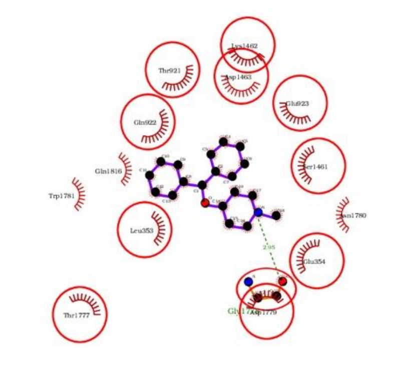
DB01148 3354 Flavoxate	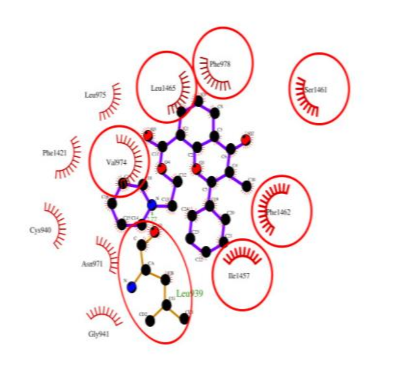	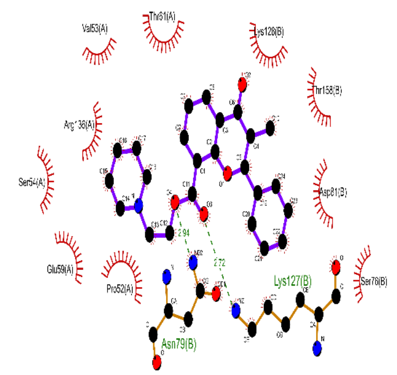	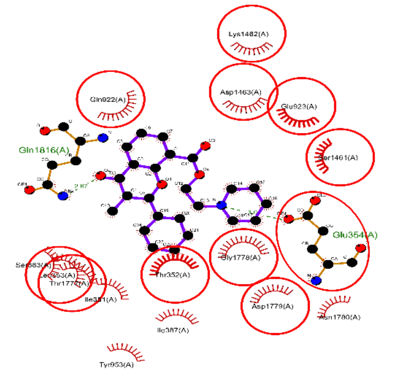
DB01501 34328 Difenoxin	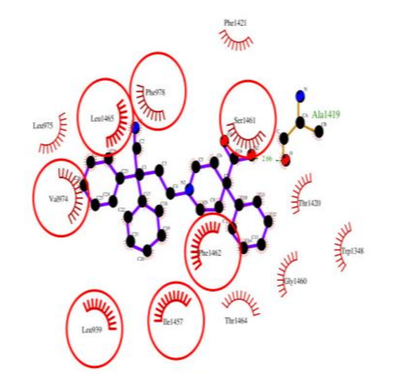	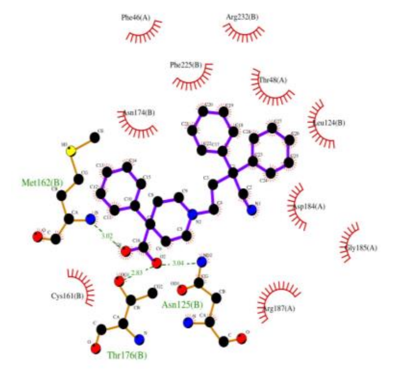	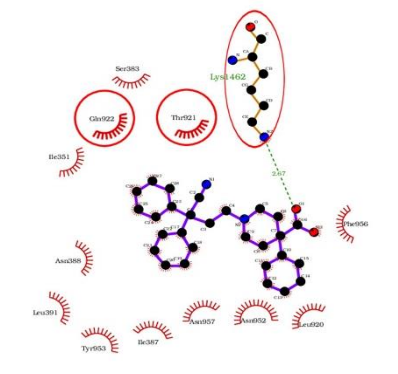
DB01544 3380 Flunitrazepam	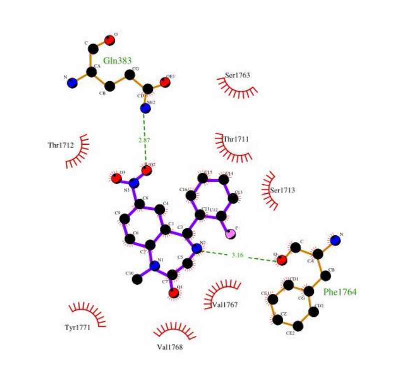	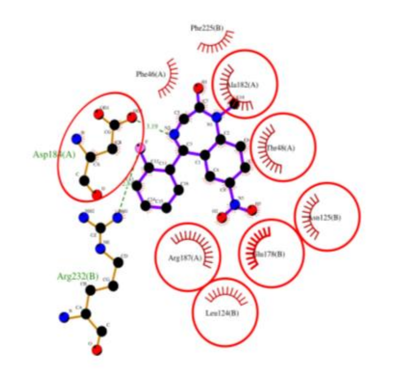	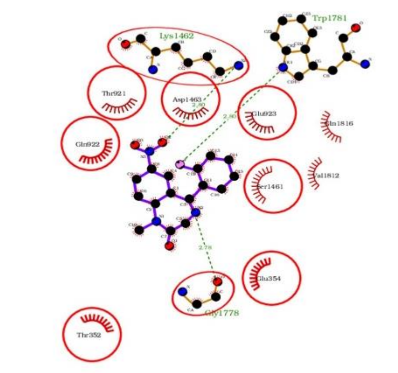
DB01608 4747 Periciazine	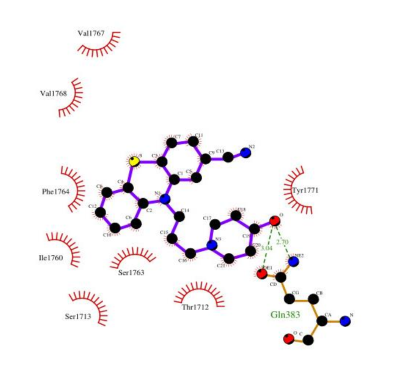	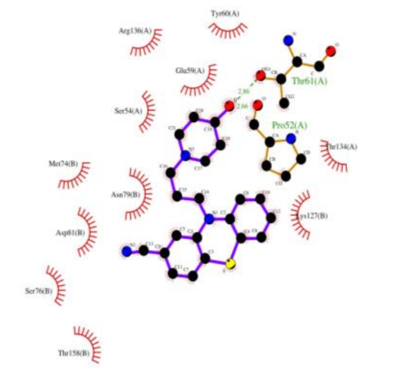	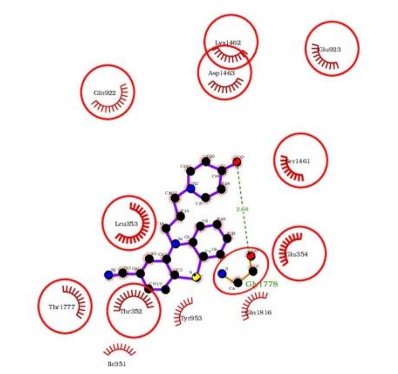

DB01623<br>941651<br>Thiothixene	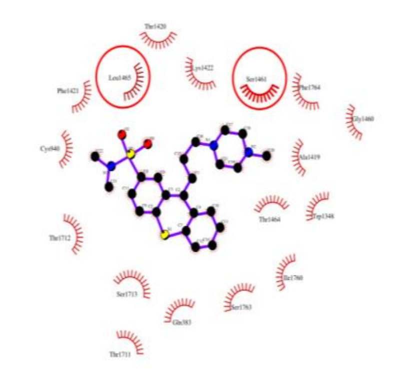	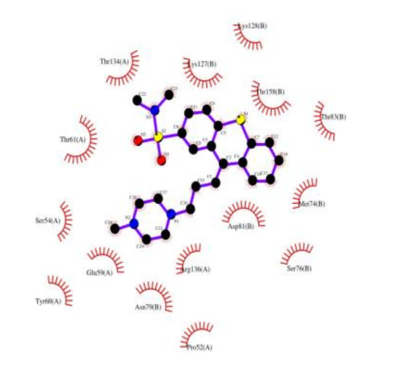	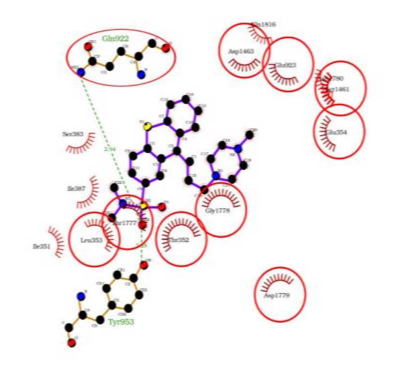
DB01624<br>5311507<br>Zuclopenthixol	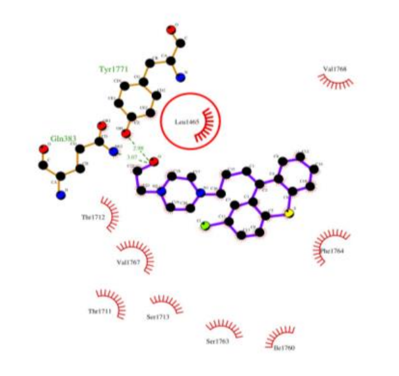	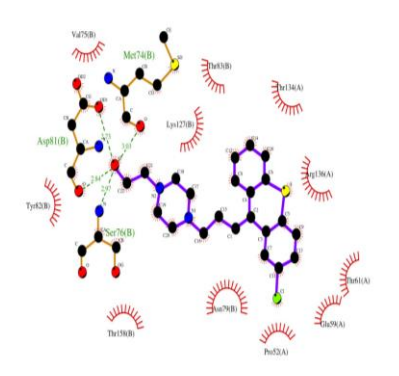	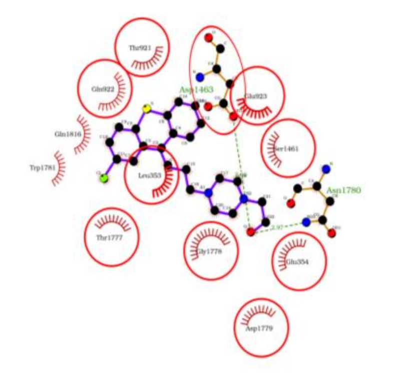
DB01628<br>123619<br>Etoricoxib	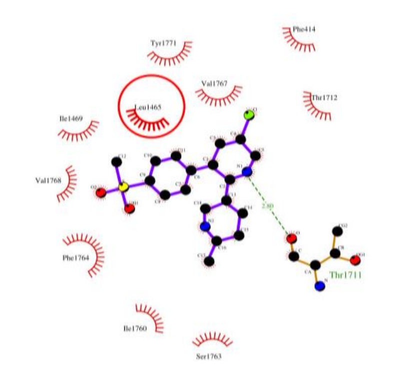	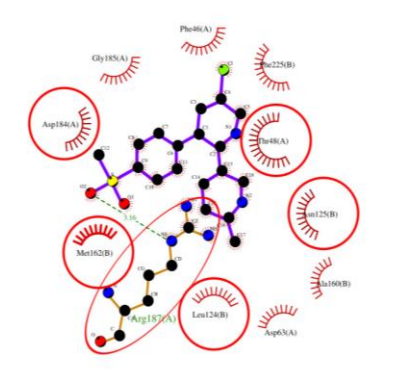	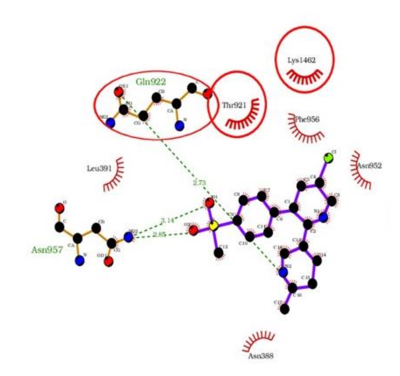
DB04841<br>941361<br>Flunarizine	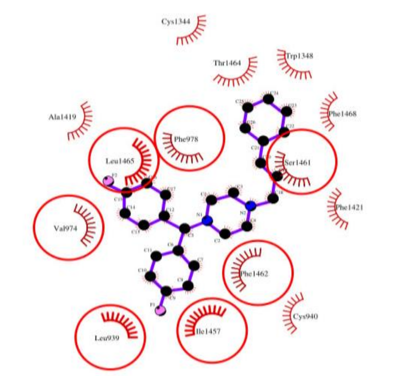	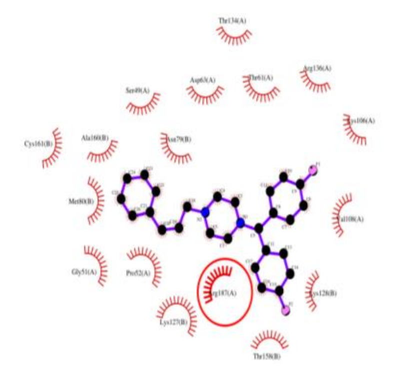	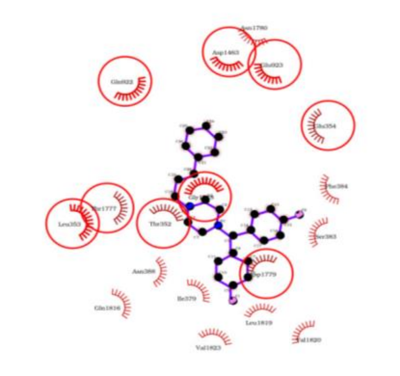
DB05015<br>6918638<br>Belinostat	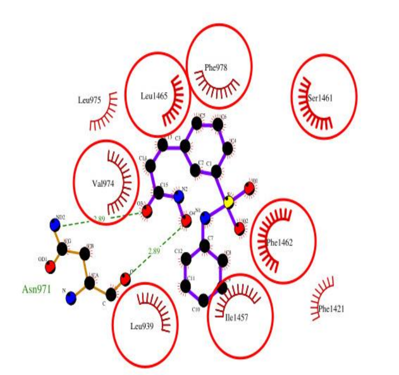	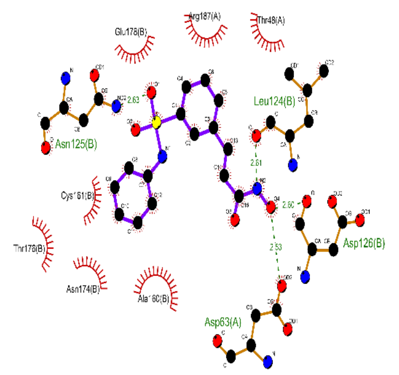	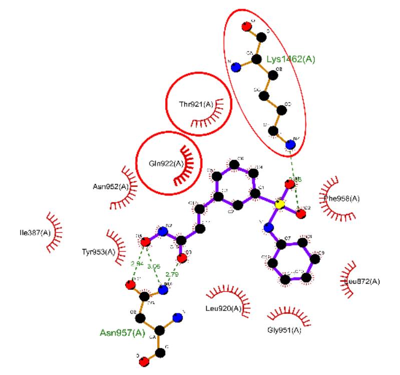

DB06077<br>21302490<br>Lumateperone	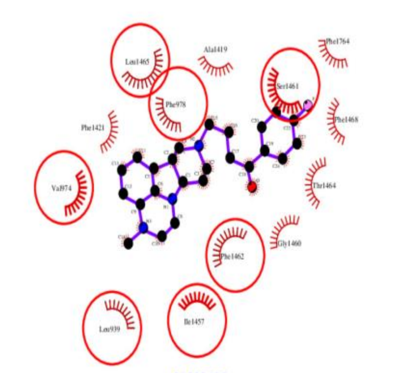	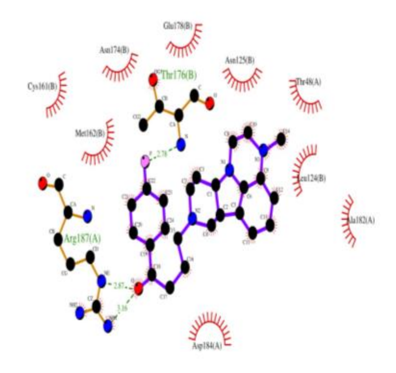	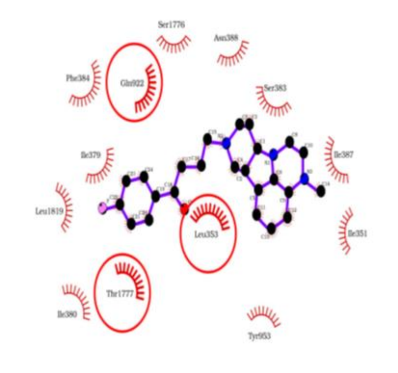
DB06153<br>27400<br>Pizotifen	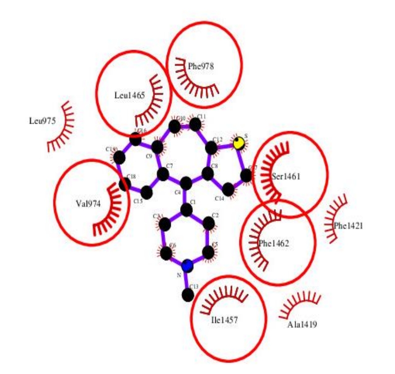	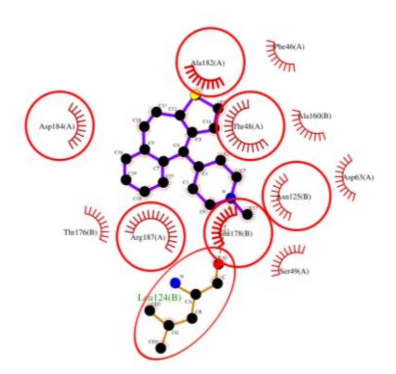	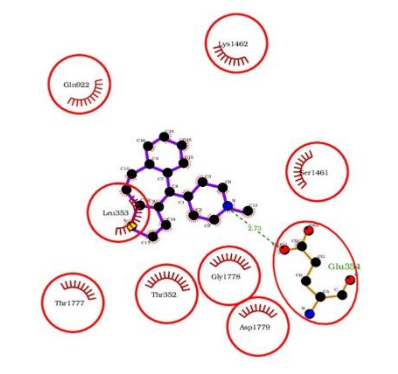
DB06413<br>9690109<br>Armodafinil	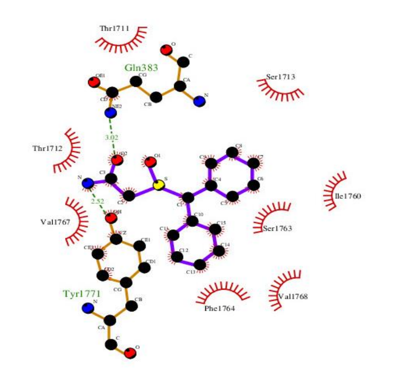	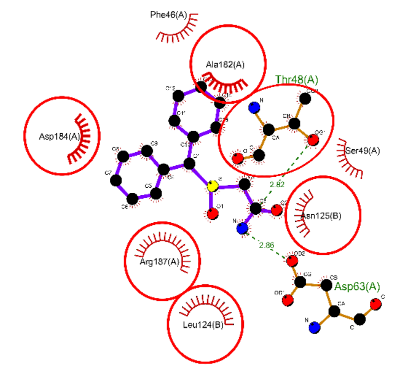	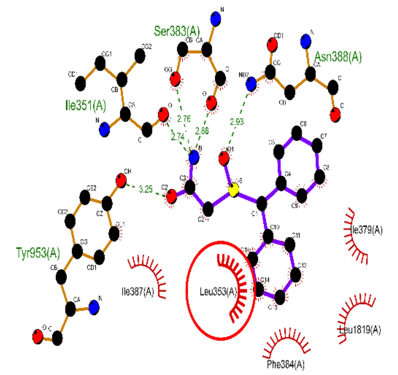
DB06626<br>6450551<br>Axitinib	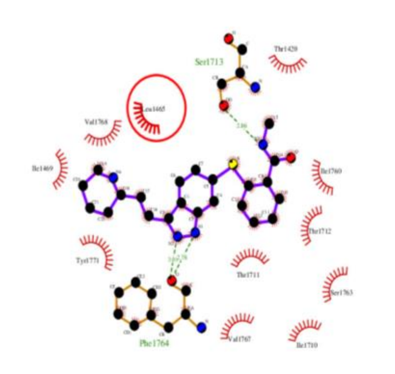	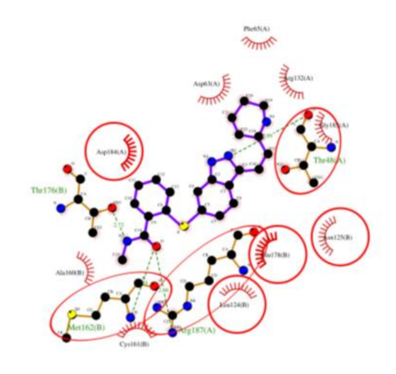	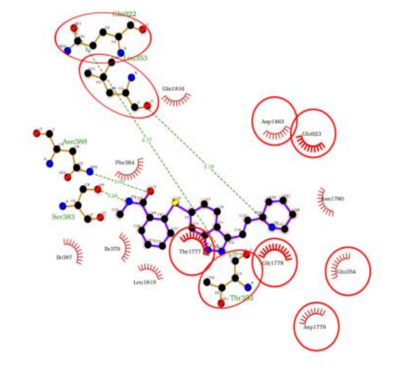
DB06684<br>6918314<br>Vilazodone	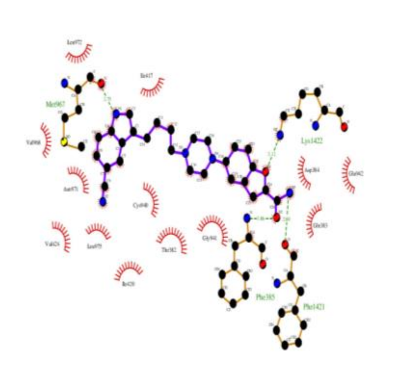	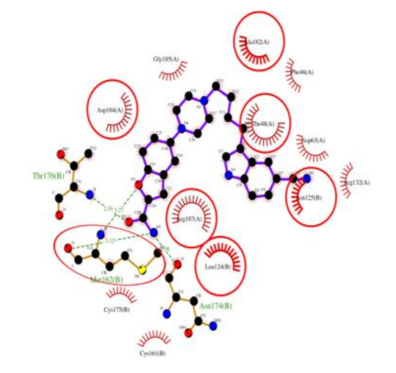	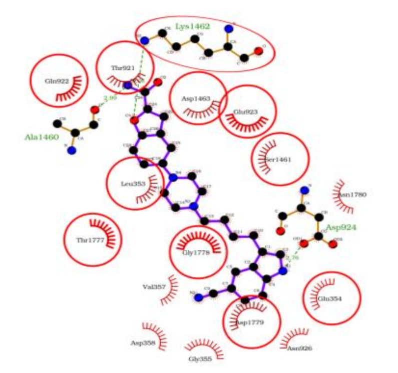

DB09167 5284550 Dosulepin	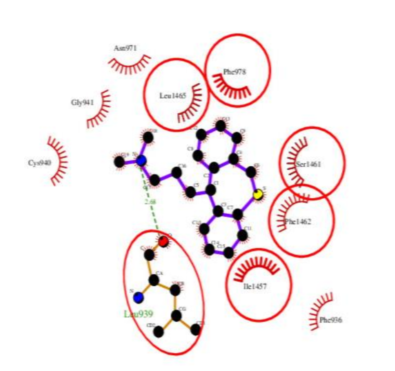	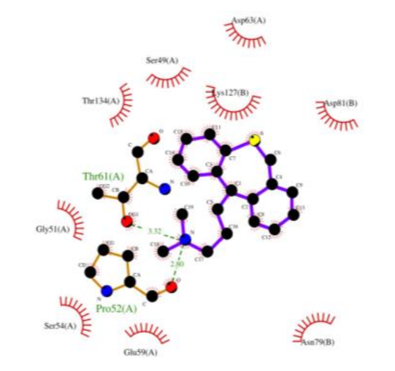	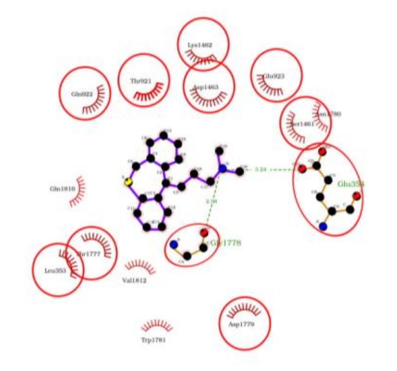
DB09488 5284514 Acrivastine	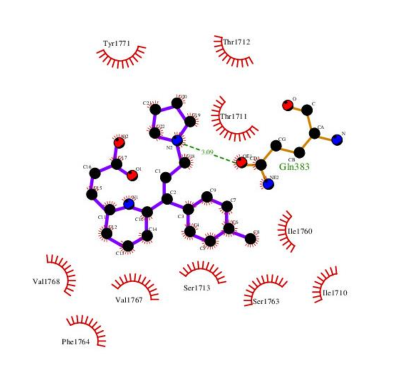	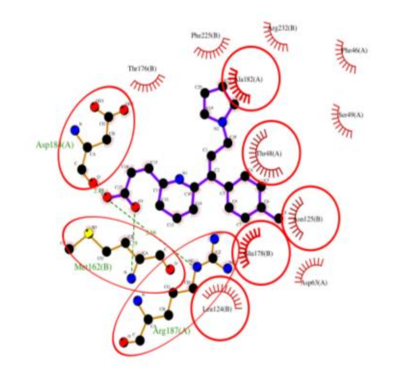	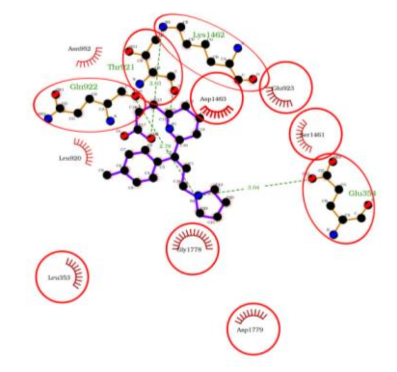
DB11952 50905713 Duvelisib	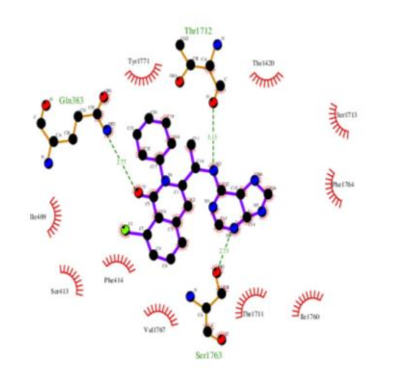	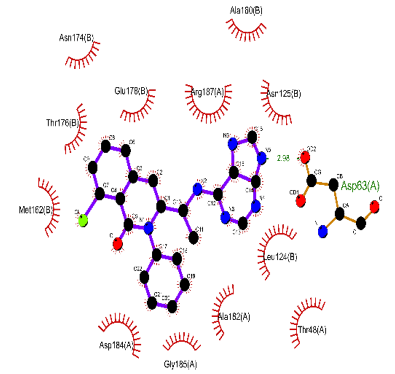	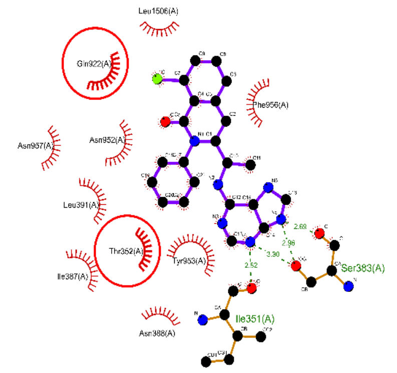
DB12492 9331 Piritramide	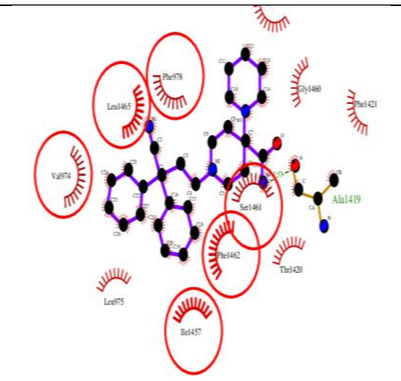	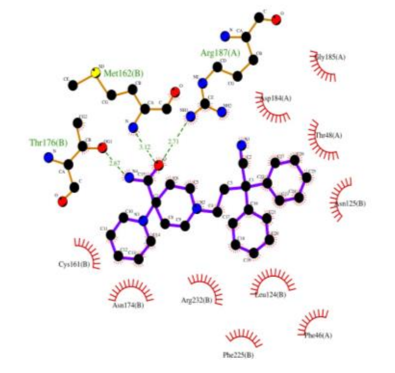	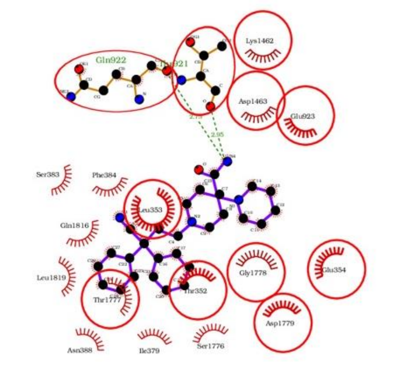
DB12792 213013 Boscalid	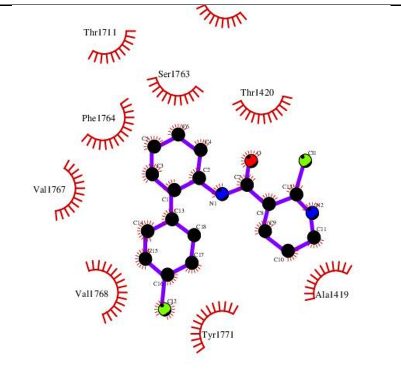	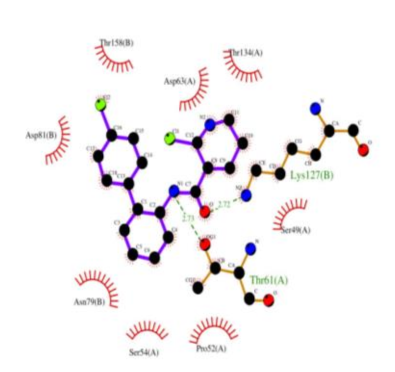	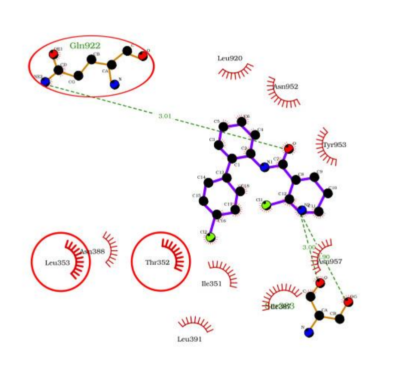

DB12877<br>4615<br>Oxatomide	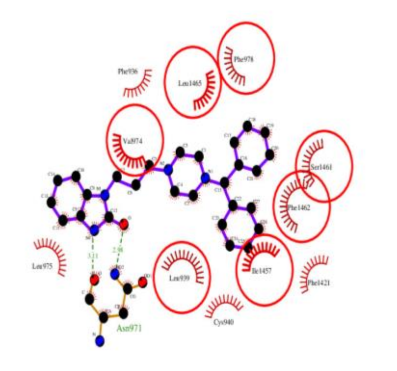	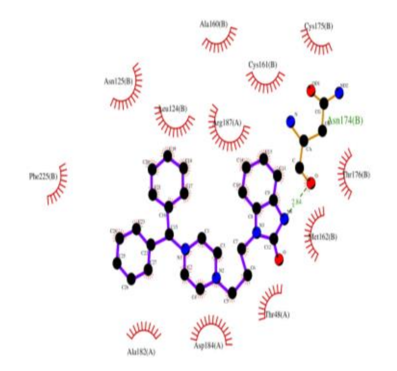	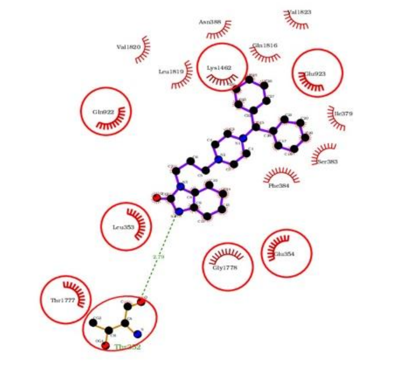
DB13292<br>4822<br>Pimethixene	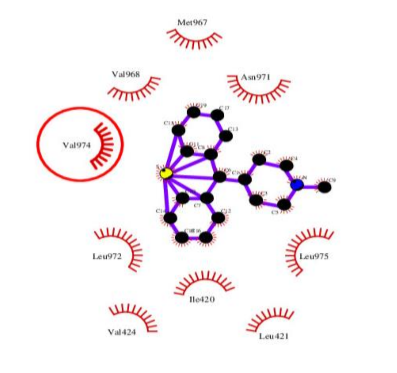	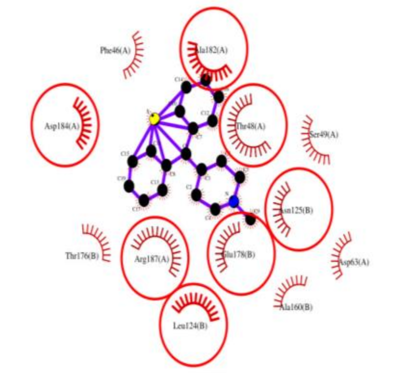	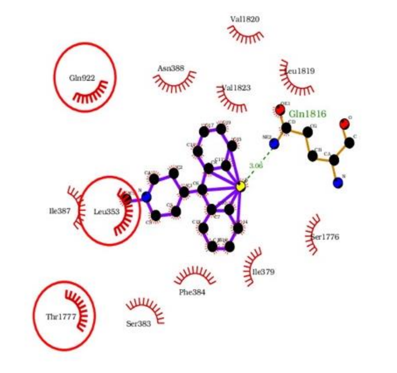
DB14033<br>6662<br>Acetyl sulfisoxazole	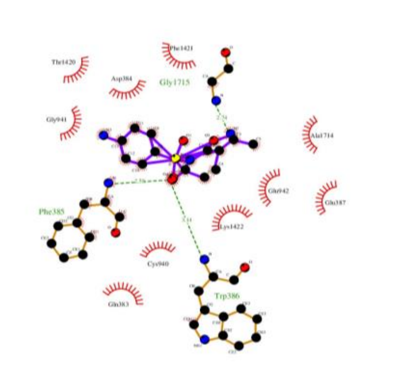	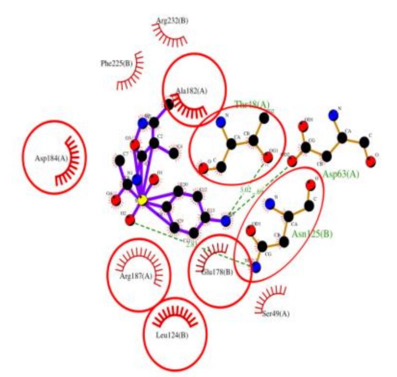	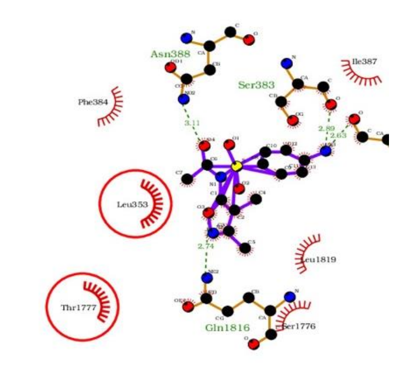
